# CoexpressDeconvolve enables reference-free single-cell-resolution deconvolution from spot-based spatial transcriptomics

**DOI:** 10.1016/j.isci.2026.116824

**Published:** 2026-09-01

**Authors:** Olga Perik-Zavodskaia, Roman Perik-Zavodskii, Saleh Alrhmoun, Sergey Sennikov

**Affiliations:** 1 Laboratory of Molecular Immunology, Research Institute of Fundamental and Clinical Immunology, Novosibirsk 630099, Russia

**Keywords:** spatial transcriptomics, Visium, reference-free deconvolution, spatial gene co-expression, single-cell reconstruction, tumor microenvironment, tongue squamous cell carcinoma, breast cancer

## Abstract

Spot-based spatial transcriptomics captures the transcriptome of multiple adjacent cells per spot, obscuring cell type-specific signals. Most deconvolution tools, therefore, depend on external single-cell references and return cell-type fractions rather than the number of cells, and only some output full expression profiles. Here we present CoexpressDeconvolve, a reference-free framework that combines a hybrid housekeeping-library-size calibration with topic modeling on a spatial gene co-expression manifold to recover integer cell counts and cell type-specific transcriptomes. Benchmarking synthetic Visium data against Tangram, cell2location, and STdeconvolve shows that CoexpressDeconvolve attains competitive expression-reconstruction fidelity, the lowest cell-count error, and the highest per-slide cell-type concordance. Our framework outputs a feature-barcode matrix that mimics standard Space Ranger output and loads directly into the standard single-cell downstream analytical stack. We applied it to human breast cancer and tongue squamous cell carcinoma, where it resolved tumor microenvironment composition and identified malignant progression axes.

## Highlights

• Reference-free reconstruction of cell type expression from spot transcriptomes
• Hybrid housekeeping and library-size calibration yields per-spot cell counts
• Reliable absolute cell counts and competitive expression fidelity in benchmarks
• Allows for the usage of downstream single-cell-tailored tools

## Graphical abstract

**Figure:**
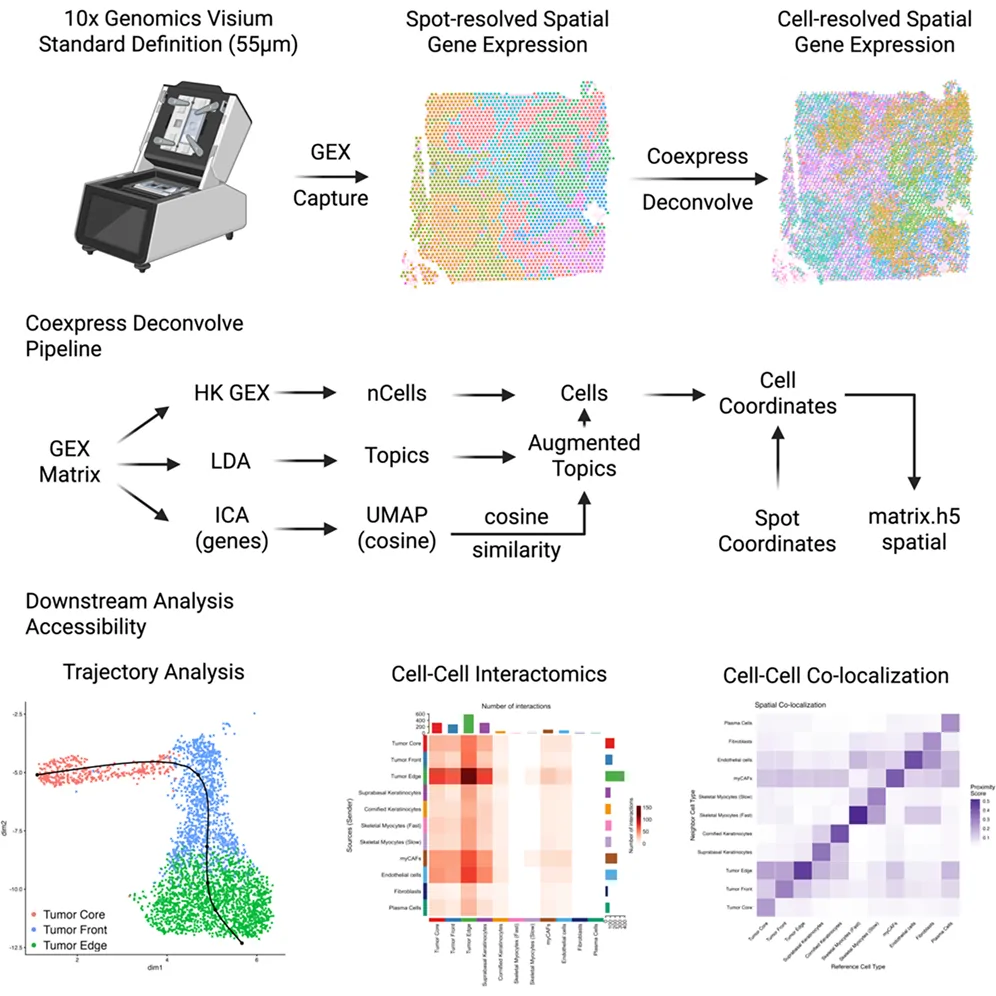

## Teaser

Genomics; Computational bioinformatics; Transcriptomics

## Introduction

Spatial transcriptomics (ST) has rapidly established itself as a cornerstone technology in modern genomics, fundamentally reshaping our understanding of tissue architecture.^1^^,^^2^^,^^3^^,^^4^ By retaining the positional context of gene expression, ST allows researchers to map the complex cellular interactions that drive biological processes ranging from the spatial organization of the tumor microenvironment in onco-immunology to the precise lineage trajectories inherent to developmental biology.^5^^,^^6^^,^^7^ This methodological leap has moved the field beyond both bulk sequencing and the dissociated abstraction of single-cell RNA sequencing (scRNA-seq), enabling integrated*, in situ* analyses that link transcriptional state to tissue context.^8^^,^^9^^,^^10^^,^^11^^,^^12^^,^^13^^,^^14^

Despite the recent emergence of sub-cellular resolution platforms such as Xenium,^15^ CosMX,^16^ and MERSCOPE,^17^ which offer the ability to pinpoint transcripts to individual cells, the field remains heavily reliant on spot-based capture technologies, most notably the 10× Genomics Visium platform. While high-resolution imaging-based methods provide exquisite detail, they are often limited by higher capital costs, lower throughput, and restricted gene panels.^15^ In contrast, spot-based ST offers an accessible, whole-transcriptome solution that integrates seamlessly with standard histological workflows. However, the multicellular character of each Visium spot creates a fundamental analytical challenge: the captured signal is a composite of several adjacent cells, requiring computational deconvolution to recover cell type-resolved measurements.^18^^,^^19^

Current deconvolution strategies are broadly categorized into reference-based and reference-free approaches. Reference-based tools, such as cell2location,^20^ RCTD,^21^ and Tangram,^22^ have become the standard for many workflows, leveraging paired scRNA-seq reference atlases to map known signatures onto spatial locations. However, these methods are inherently constrained by the availability and quality of the reference data; in scenarios involving novel pathological states or organisms lacking robust atlases, they risk misidentification or the hallucination of absent cell types. Consequently, reference-free methods such as STdeconvolve^23^ have emerged as a complementary direction.

While methods like STdeconvolve can identify cell types without external reference data, they retain limitations that restrict downstream usage. First, these pipelines typically return relative cell-type proportions rather than absolute cellular abundance. Second, many existing approaches rely on a small set of over-dispersed genes (ODGs) for computational efficiency, which discards the majority of the transcriptome and loses the low-expressed markers needed for fine cell-type identification. Finally, results are often presented as abstract probabilistic topics rather than discrete single-cell-like profiles, complicating integration with standard single-cell analytical pipelines.

To address these issues, we developed CoexpressDeconvolve, a reference-free pipeline that reconstructs cell type-specific gene expression directly from spot-based spatial transcriptomic data. Our method first approximates the number of cells in each spot through a hybrid calibration that combines a housekeeping (HK) gene signal with the spot’s total library size, yielding a single estimator that remains stable across transcriptionally variable regions. To recover the full transcriptome, it then uses fast independent component analysis (FastICA) to build a spatial gene co-expression manifold that acts as a scaffold for projecting the limited ODG set onto the whole transcriptome by identifying geometric neighbors in the co-expression space. The pipeline employs latent Dirichlet allocation (LDA)^24^ to define cell-type topics and finally distributes the observed unique molecular identifiers (UMIs) of each spot into discrete single-cell-like profiles via hierarchical Bayesian sampling. In this work, we benchmark CoexpressDeconvolve against the reference-based methods Tangram and cell2location and the reference-free method STdeconvolve on synthetic Visium data with known ground truth, apply it to a real human breast cancer slide with a paired scRNA-seq atlas, and demonstrate its utility on a tongue squamous cell carcinoma slide where it resolves the tumor progression axis architecture and signaling network.

## Results

### A reference-free benchmark for spot-level deconvolution

**Figure 1:**
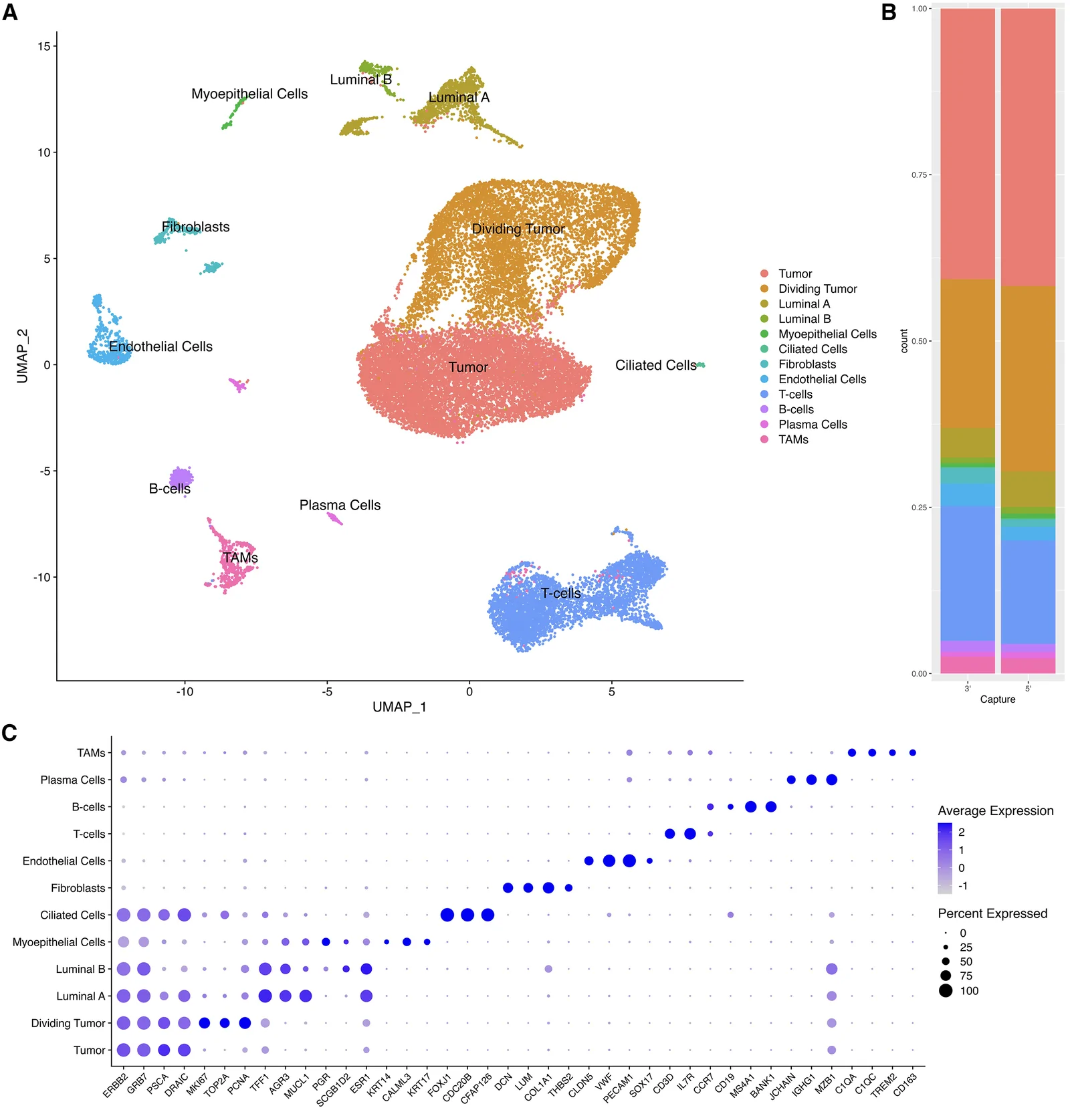
Ground-truth breast cancer scRNA-seq atlas for spatial deconvolution benchmarking (A) UMAP embedding of integrated 3′ and 5′ end scRNA-seq breast cancer datasets, colored by annotated cell type. (B) Relative cell-type proportions across the 3′ and 5′ capture chemistries. (C) Dot plot of canonical marker genes used for cell-type assignment.

To establish a biologically matched reference for benchmarking, we used publicly available human breast cancer datasets generated by 10× Genomics, in which scRNA-seq and Visium spatial transcriptomics were profiled from paired sections of the same specimen (Figures 1A–1C). Integration of the 3′ and 5′ end scRNA-seq libraries yielded a transcriptionally coherent atlas encompassing the principal epithelial, stromal, endothelial, and immune compartments of breast cancer tissue (Figure 1A). Malignant epithelial cells formed a dominant cluster that segregated into cycling (dividing tumor) and non-cycling (tumor) states, while non-malignant epithelial populations resolved into luminal A, luminal B, myoepithelial, and ciliated cells. Stromal populations (fibroblasts and endothelial cells) were clearly separated from epithelial clusters, and immune populations (T cells, plasma cells, and tumor-associated macrophages [TAMs]) formed lineage-consistent clusters.

Modest shifts in relative proportions were observed between chemistries, but epithelial, stromal, and immune compartments were reproducibly recovered (Figure 1B), indicating the robustness of the transcriptional profiles independent of library preparation strategy. Marker gene analysis confirmed canonical lineage identities and high transcriptional specificity (Figure 1C). This cross-chemistry consistency established the suitability of the integrated dataset as a stable ground-truth reference for downstream synthetic and real-data benchmarking.

### CoexpressDeconvolve reconstructs cell type-specific gene expression at par with reference-based methods

To quantitatively compare CoexpressDeconvolve against state-of-the-art deconvolution tools, we constructed a synthetic Visium dataset from the breast cancer scRNA-seq atlas (see STAR Methods). Each of 2,500 simulated spots contained 1–10 cells sampled with replacement from the atlas, providing a known ground truth for both cell-type composition and cell type-specific gene expression. We ran CoexpressDeconvolve alongside two reference-based methods (Tangram^22^ and cell2location^20^) and one reference-free method (STdeconvolve^23^) on the same input matrices, using identical ODG sets and the same atlas as reference.

**Figure 2:**
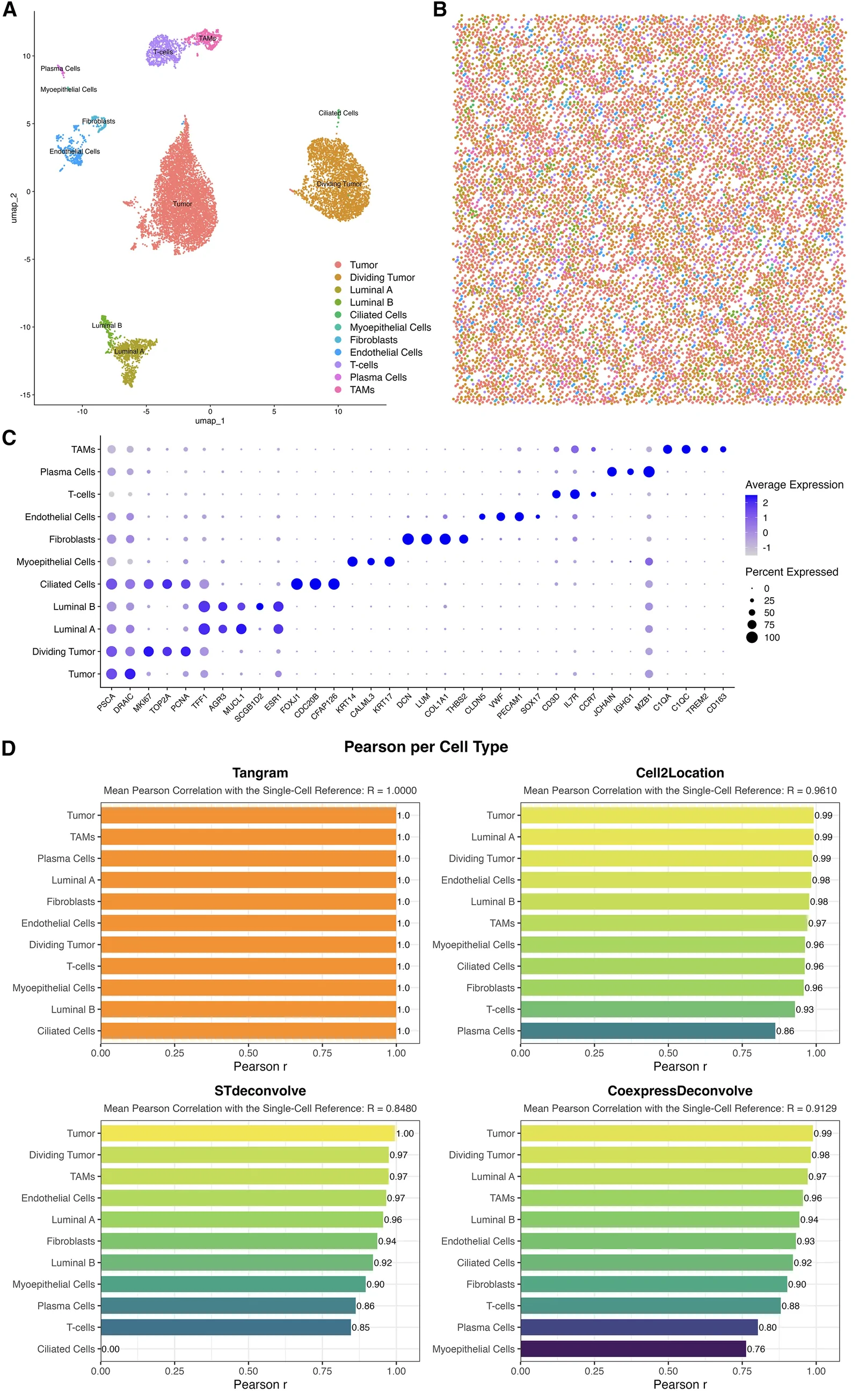
Benchmarking of cell type-specific gene expression reconstruction on synthetic Visium data (A) UMAP of CoexpressDeconvolve single-cell-like profiles inferred from the synthetic Visium spots. (B) Spatial projection of the inferred populations onto the synthetic spot grid. (C) Dot plot of canonical marker genes in the CoexpressDeconvolve reconstruction. (D) Per-cell-type Pearson correlation of pseudo-bulk profiles with the matched single-cell ground truth for Tangram, cell2location, STdeconvolve, and CoexpressDeconvolve, computed across 3,000 highly variable genes. Top-right numerical labels indicate the mean Pearson correlation across cell types.

Following deconvolution, CoexpressDeconvolve recovered sharply delineated, lineage-consistent clusters in low-dimensional space (Figure 2A). Spatial remapping of the reconstructed populations preserved the expected structure of the synthetic spots (Figure 2B), and canonical epithelial, proliferation, stromal, endothelial, and immune signatures were strongly enriched within their expected reconstructed populations with minimal cross-lineage signal leakage (Figure 2C).

To quantitatively evaluate reconstruction fidelity, we compared the per-cell-type pseudo-bulk profiles of each method to the ground-truth scRNA-seq populations across 3,000 highly variable genes by using the Pearson correlation coefficient and Lin’s concordance correlation coefficient (CCC) (Figure 2D). As expected by construction, Tangram approached perfect agreement with the reference (mean Pearson r = 1.000, CCC = 1.000), since in cells mode it returns the reference expression weighted onto each spot. Among the remaining three methods, cell2location achieved the highest fidelity (mean Pearson r = 0.961, CCC = 0.942), followed by CoexpressDeconvolve (mean Pearson r = 0.913, CCC = 0.905) (Figure S1) and STdeconvolve (mean Pearson r = 0.848, CCC = 0.828). CoexpressDeconvolve, therefore, outperformed STdeconvolve on the gene expression reconstruction task by 0.06 Pearson units and trailed cell2location by approximately 0.05 Pearson units, despite not using the single-cell reference at any stage.

### Absolute cell counts and cell-type proportion accuracy across methods

**Figure 3:**
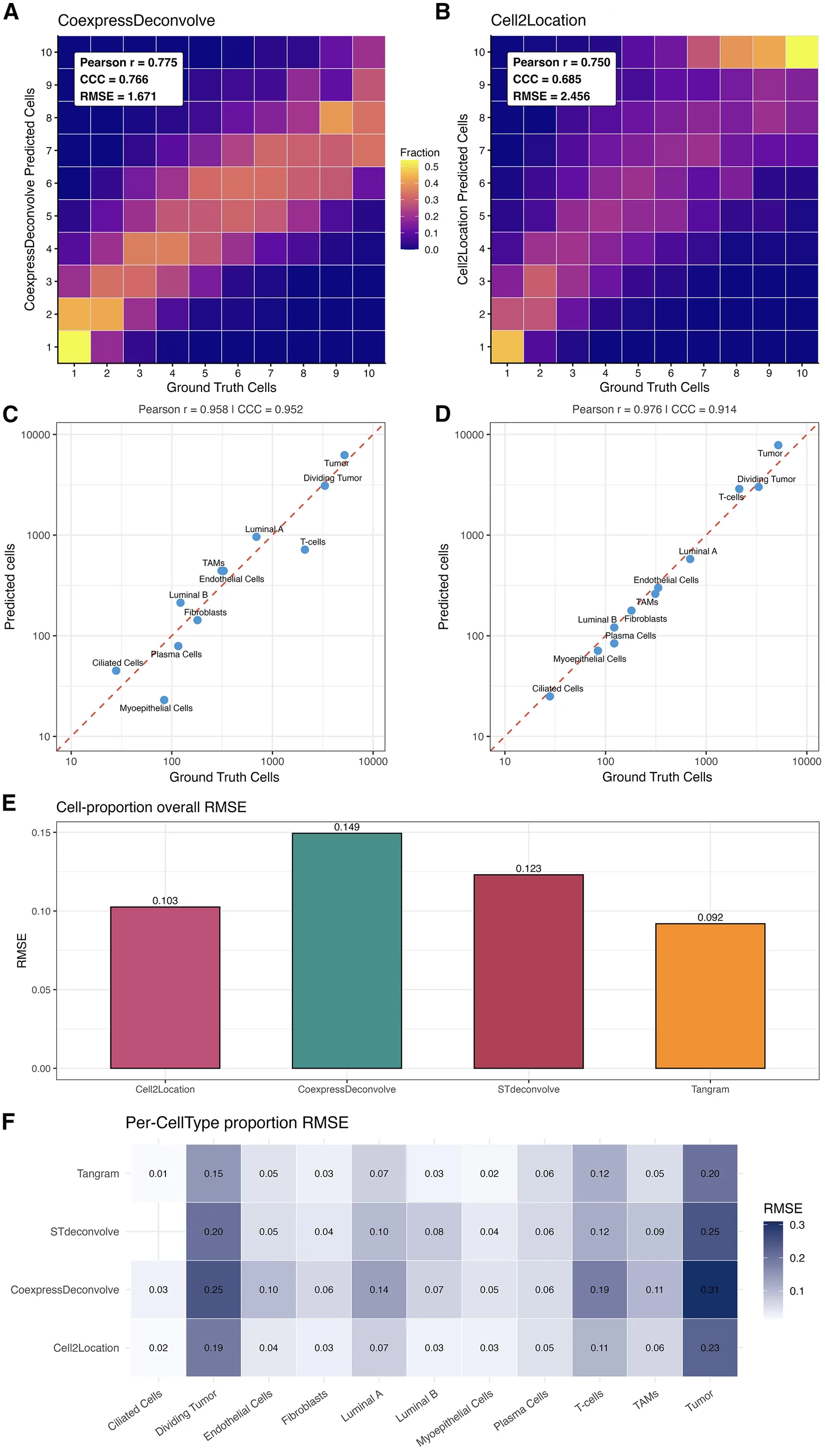
Cell-count and cell-type proportion benchmarking on synthetic Visium data (A and B) Two-dimensional density of predicted versus ground-truth total cell count per spot for CoexpressDeconvolve (A) and cell2location (B); Pearson, CCC, and RMSE statistics are annotated, yellow depicts high number of cells per bin, purple depicts no cells in a bin. (C and D) Per-cell-type slide-level cell totals for CoexpressDeconvolve (C) and cell2location (D); each point is one cell type. (E) Overall cell-type proportion RMSE for cell2location, CoexpressDeconvolve, STdeconvolve, and Tangram. (F) Per-cell-type proportion RMSE heatmap across the four methods; the tumor/dividing tumor pair is the consistent error driver.

Beyond gene expression fidelity, a key practical question is whether a method recovers the actual number of cells per spot and the cell-type composition of the slide. Only CoexpressDeconvolve and cell2location return per-spot integer cell counts; Tangram and STdeconvolve return relative weights or proportions. We, therefore, split the cell-quantification benchmark into a head-to-head count comparison (CoexpressDeconvolve versus cell2location) and a four-way proportion-RMSE (root-mean-square error) comparison (Figure 3).

On per-spot total cell counts, CoexpressDeconvolve produced predictions that tracked the ground truth with a Pearson correlation of 0.775, CCC of 0.766, and RMSE of 1.671 cells, while slightly underestimating spots with 8–10 cells (Figure 3A). cell2location attained a Pearson correlation of 0.750 and CCC of 0.685 with an RMSE of 2.456 cells, with a tendency to overestimate cell number across the count range (Figure 3B). On the aggregated per-cell-type cell totals across the slide, CoexpressDeconvolve again outperformed cell2location (Pearson 0.775 vs. 0.750, CCC 0.952 vs. 0.914; Figures 3C and 3D), driven by the better-calibrated per-spot estimates. Quantitatively, CoexpressDeconvolve recovered the absolute number of cells per spot at 0.68 times the error of cell2location, providing single-cell-resolution counts without the need for an external reference and a supplied implied mean of the cells per spot, like cell2location does.

On cell-type proportions, however, CoexpressDeconvolve finished last among the four methods (overall RMSE 0.149), trailing Tangram (0.092), cell2location (0.103), and STdeconvolve (0.123) (Figure 3E). The gap was modest but consistent, and reflects the trade-off intrinsic to a reference-free framework: without a curated single-cell signature, the model must infer cell-type identities purely from the topology of the spatial gene co-expression manifold, which is harder for cell types whose transcriptomes differ only by a small set of cycling or context genes.

A per-cell-type proportion RMSE heatmap (Figure 3F) revealed a method-agnostic confounder: tumor and dividing tumor cells were the hardest pair for all four pipelines. These two states differ primarily by an S/G2M-phase program superimposed on an otherwise shared malignant transcriptome, and were the leading contributors to the proportion error across every method. Other cell types with substantial transcriptional individuality were recovered with proportion RMSE values within a narrow band across methods. We, therefore, interpret CoexpressDeconvolve as offering the best absolute cell quantification and competitive expression fidelity, at the cost of a small absolute proportion penalty driven primarily by the tumor/dividing tumor confusion shared by all benchmarked tools.

### Application to real human breast cancer Visium tissue

**Figure 4:**
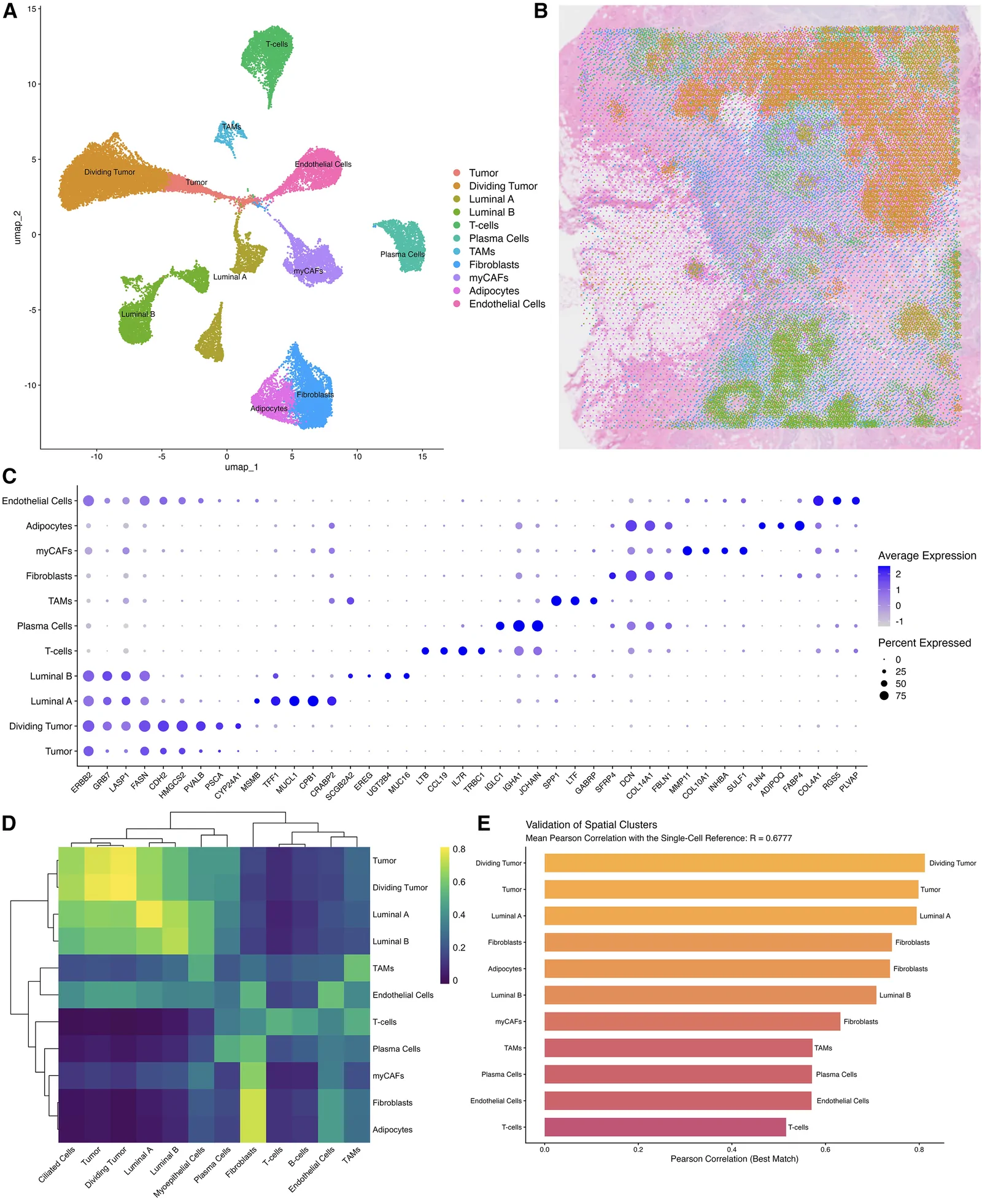
CoexpressDeconvolve recovers atlas-consistent cellular programs in a real breast cancer Visium slide (A) UMAP embedding of CoexpressDeconvolve single-cell-like profiles inferred from the breast cancer Visium slide. (B) Spatial projection of the inferred populations onto the histological section. (C) Dot plot of canonical marker genes. (D) Hierarchical clustering heatmap of cluster-averaged Pearson correlations with the matched scRNA-seq reference, computed across 3,500 highly variable genes, yellow depicts high gene expression correlation, purple depicts no gene expression correlation. (E) Per-cluster Pearson correlation with the best-matching single-cell reference population.

We next applied CoexpressDeconvolve to the real human breast cancer Visium slide paired to the scRNA-seq atlas described earlier (Figure 4A). The reconstructed uniform manifold approximation and projection (UMAP) exhibited sharply separated populations corresponding to tumor, dividing tumor, luminal A, luminal B, myoepithelial cells, T cells, plasma cells, TAMs, fibroblasts, myCAFs, adipocytes, and endothelial cells (Figure 4A), and the spatial layout preserved coherent tumor-stroma boundaries on the H&E section (Figure 4B).

Marker gene analysis confirmed canonical lineage-specific expression with minimal cross-cluster leakage; malignant populations showed enrichment of proliferation-associated genes, while luminal, myoepithelial, fibroblast, and immune subsets expressed their respective cell type-defining markers (Figure 4C). Notably, the deconvolved populations included Adipocytes (Figure S2) and myCAFs that are typically lost or under-represented in dissociation-based scRNA-seq, illustrating how a reference-free framework can recover cell types absent from a paired atlas.

To quantify reconstruction fidelity on real tissue, we computed Pearson correlations between the CoexpressDeconvolve cluster-averaged profiles and the matched scRNA-seq reference across 3,500 highly variable genes (Figures 4D, 4E, and S3). Hierarchical clustering of averaged profiles demonstrated clear alignment between the spatially resolved populations and their single-cell counterparts (Figure 4D). The mean Pearson correlation across cell types was 0.67 on the real slide (Figure 4E), with the highest values for malignant populations and lower values for less abundant immune and stromal populations. We note that this correlation value is lower than the 0.91 reported on the synthetic benchmark, consistent with the well-documented gap between biologically and technically non-identical scRNA-seq and Visium RNA capture protocols and rates.

### Spatial architecture and tumor invasion dynamics in tongue squamous cell carcinoma

**Figure 5:**
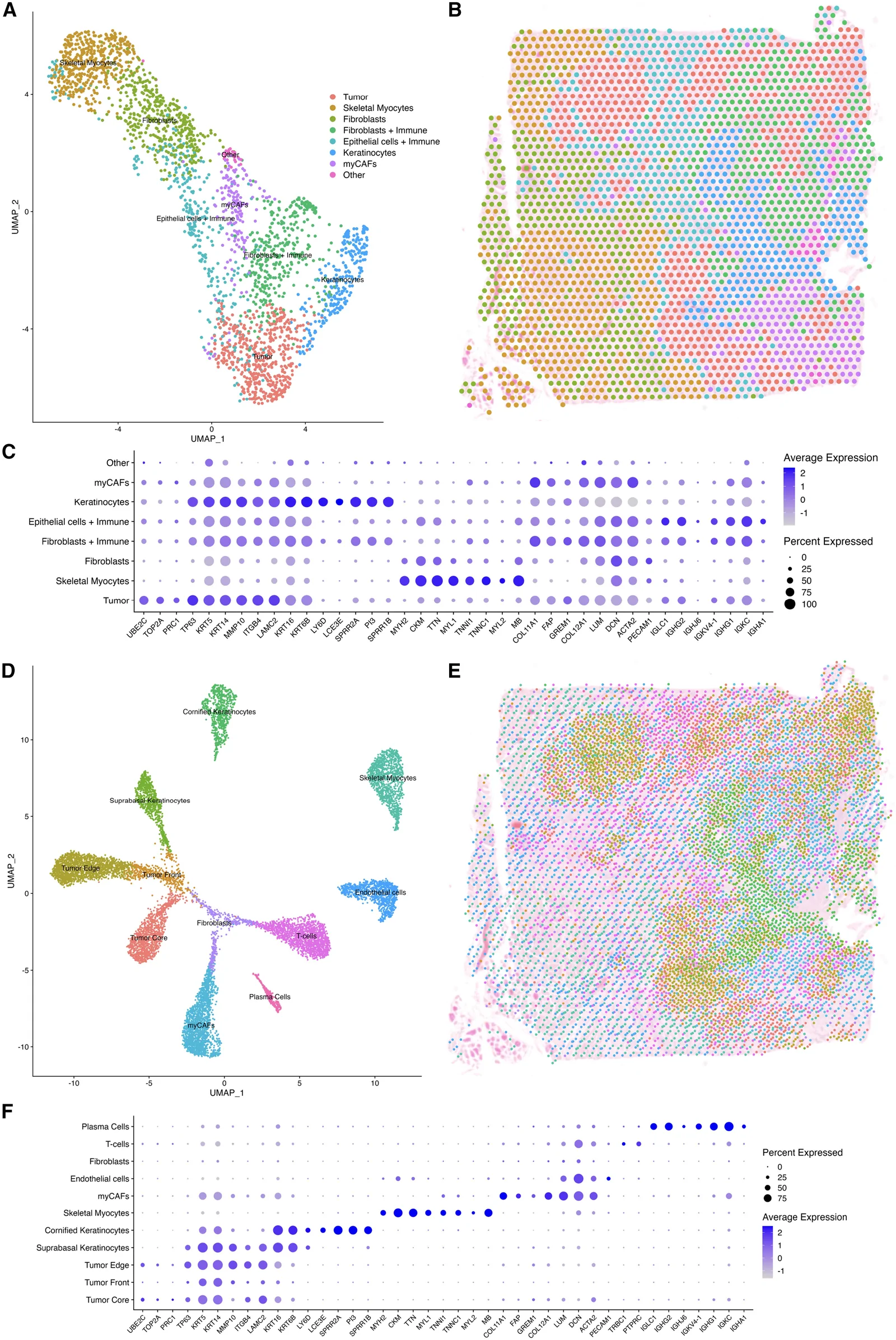
Spatial transcriptomic profiling of tongue squamous cell carcinoma before and after CoexpressDeconvolve (A) UMAP of Visium spot-level transcriptomes before deconvolution. (B) Spatial projection of the pre-deconvolution clusters onto the histological section. (C) Dot plot of marker genes for the pre-deconvolution clusters. (D) UMAP of CoexpressDeconvolve single-cell-like profiles after deconvolution. (E) Spatial projection of the reconstructed populations onto the histological section. (F) Dot plot of marker genes for the post-deconvolution populations.

To demonstrate the real-world utility of CoexpressDeconvolve in a setting where a high-quality paired scRNA-seq reference is not available, we applied it to a human tongue squamous cell carcinoma Visium dataset^2^ (Figure 5). Clustering of the raw Visium spot-level transcriptomes revealed broad transcriptional domains corresponding to tumor cells, fibroblasts, immune-associated stromal populations, keratinocytes, skeletal myocytes, and myofibroblasts (Figure 5A). However, because each Visium spot captures transcripts from multiple adjacent cells, the resulting embedding reflected composite transcriptional states rather than discrete cell types, with spots scattered along axes of mixed identity (Figures 5B and 5C).

Following CoexpressDeconvolve reconstruction, the transcriptional manifold underwent a pronounced reorganization, recovering orthogonal lineage axes (Figure 5D). The post-deconvolution UMAP showed sharply separated clusters corresponding to tumor core, tumor front, tumor edge, suprabasal keratinocytes, cornified keratinocytes, fibroblasts, myCAFs, endothelial cells, skeletal myocytes, plasma cells, and T cells. Spatial remapping of these reconstructed populations exhibited anatomically coherent organization (Figure 5E): stromal niches formed organized compartments separate from skeletal muscle regions, immune-enriched areas localized to defined spatial domains rather than appearing diffusely distributed, tumor core populations occupied compact central regions, and tumor front and tumor edge cells aligned specifically with stromal and immune interfaces. Differentiated keratinocyte populations displayed the layered spatial organization expected of native epithelium (Figure 5E). Marker gene analysis on the deconvolved populations confirmed lineage-specific expression with substantially reduced cross-cluster bleeding compared with the pre-deconvolution clusters (Figures 5C–5F).

**Figure 6:**
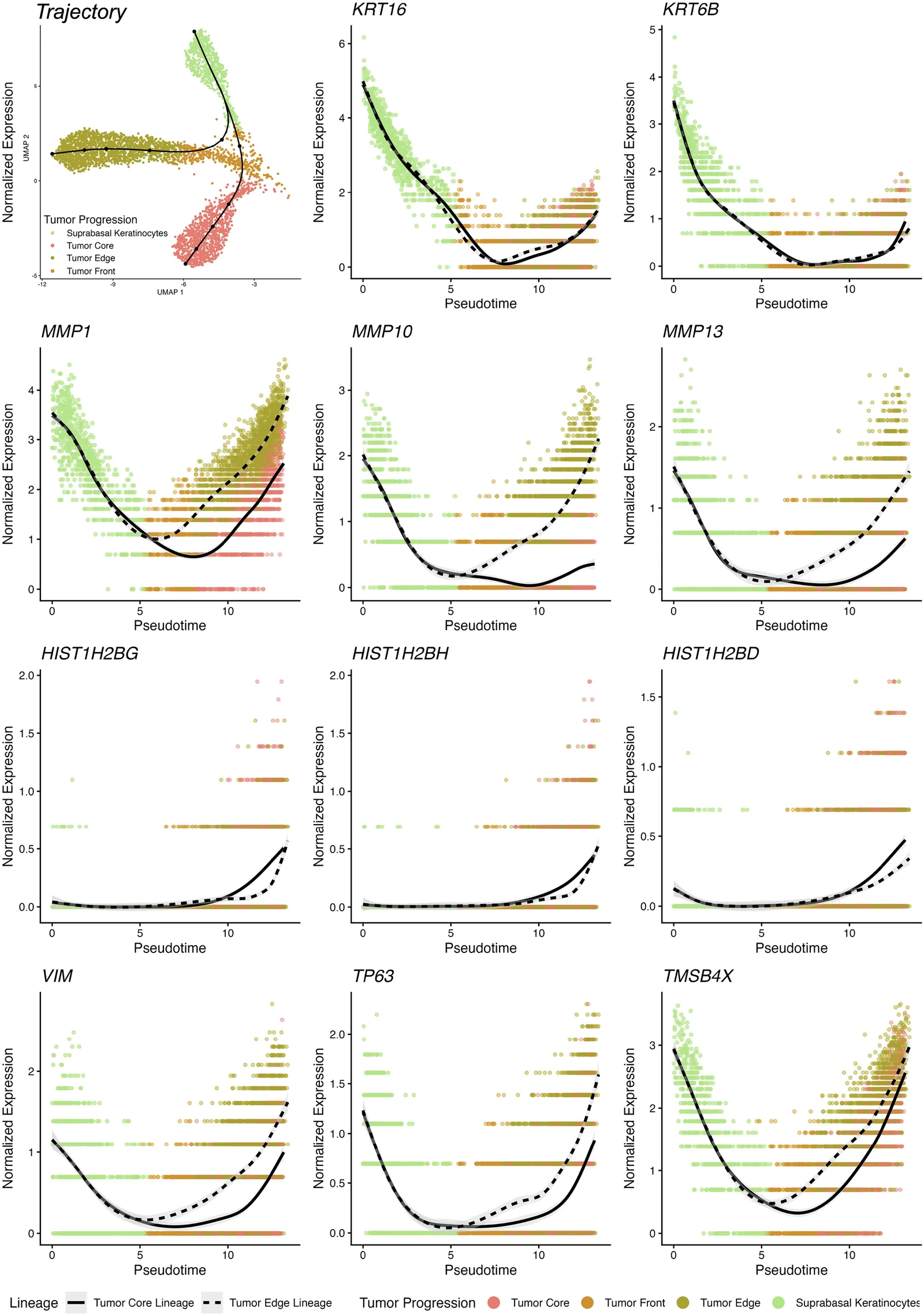
Pseudotemporal dynamics along the suprabasal keratinocyte-tumor core/tumor edge bifurcation Top, left: UMAP of the malignant compartment colored by spatial annotation (suprabasal keratinocytes, green; tumor core, red; tumor front, blue; tumor edge, green) with the two principal curves inferred by Slingshot. The remaining images show GAM-smoothed expression of representative genes along the two pseudotemporal lineages.

To delineate the transcriptional dynamics driving tumor progression, we applied trajectory inference to the reconstructed malignant compartment together with the suprabasal keratinocyte population. The inferred trajectory anchored on suprabasalkKeratinocytes and bifurcated into two malignant lineages: a tumor core lineage characterized by proliferation programs (*HIST1H2BG*, *HIST1H2BH*, and *HIST1H2BO*) and a tumor edge lineage marked by epithelial-mesenchymal transition and matrix-remodeling programs (*MMP1*, *MMP10*, *MMP13*, *VIM*, *TP63*, and *TMSB4X*) (Figure 6).^25^ The expression of cytokeratins *KRT16* and *KRT6B* was sharply reduced as cells transitioned out of the suprabasal keratinocyte ancestor toward both malignant fates, while *MMP* family genes and *VIM* rose specifically along the tumor edge branch, consistent with a coordinated invasive program.

### Intercellular communication at the invasive edge

**Figure 7:**
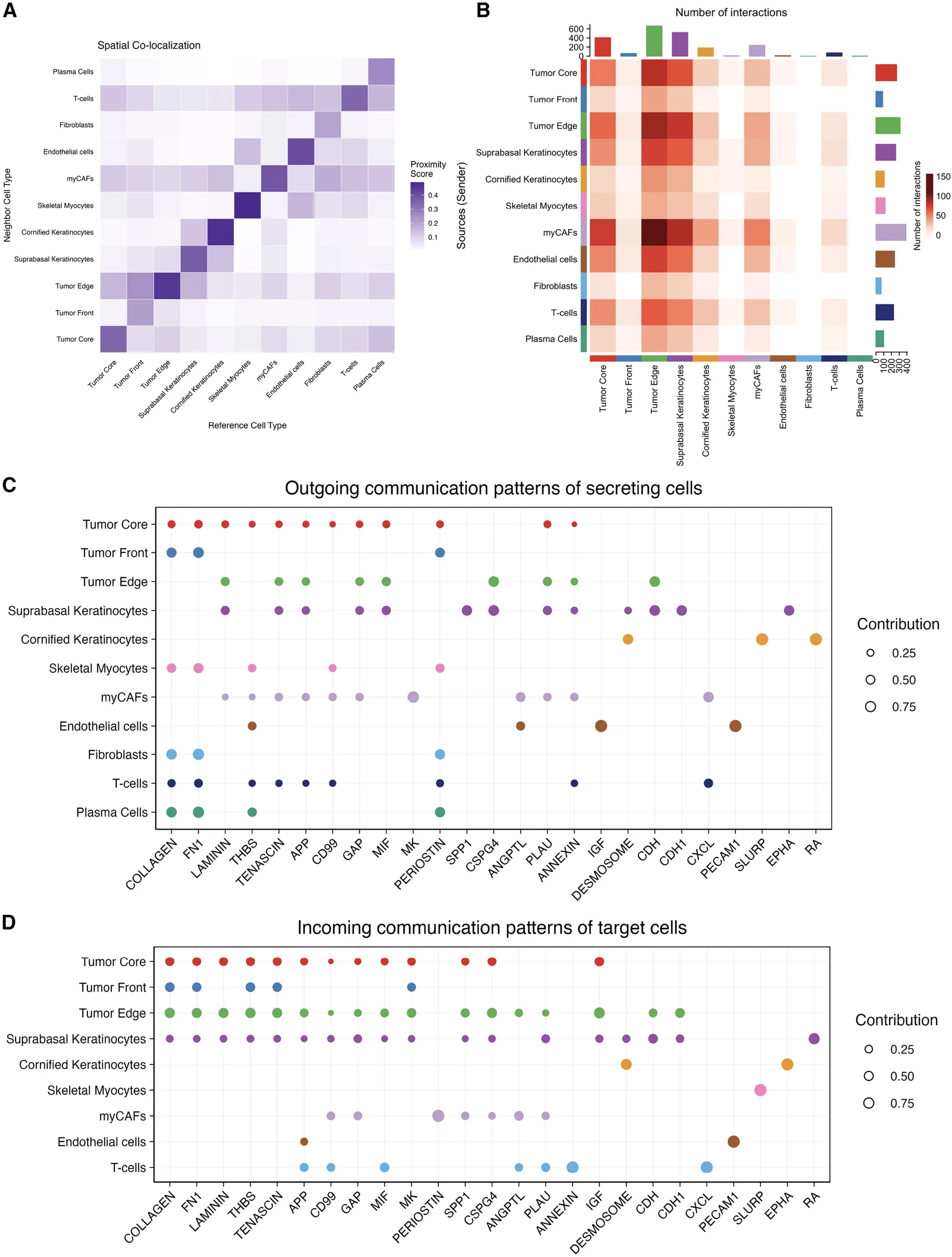
Predicted intercellular communication network in tongue squamous cell carcinoma (A) Heatmap of pairwise spatial co-localization scores across CoexpressDeconvolve-inferred cell types, purple depicts high spatial proximity score, white depicts low spatial proximity score. (B) Heatmap of the inferred number of ligand-receptor interactions between cell-type pairs; the tumor edge dominates outgoing and incoming interactions, deep red depicts high number of interactions, white depicts no interatctions. (C) Dot plot of dominant outgoing signaling pathways across secreting cell types; dot size indicates relative contribution to pathway activity. (D) Dot plot of incoming signaling pathways for target cell types.

The ability to reconstruct cell type-specific gene expression from spot data enabled us to apply downstream single-cell analytical tools, in particular CellChat,^26^ to dissect intercellular communication within the spatial context (Figure 7). Spatial co-localization analysis of the reconstructed populations revealed a structured, compartmentalized tissue organization (Figure 7A). Malignant populations formed a coherent spatial module (tumor core, tumor front, and tumor edge) with high mutual proximity, consistent with a spatially ordered malignant continuum. This malignant axis was spatially associated with suprabasal keratinocytes, reflecting epithelial contiguity, whereas stromal components (fibroblasts, myCAFs, and endothelial cells) formed a distinct colocalization block.

The CellChat-inferred number of ligand-receptor interactions revealed that tumor edge and myCAFs were the dominant communication hubs, displaying the highest overall number of outgoing and incoming interactions (Figure 7B). Cornified keratinocytes and skeletal myocytes exhibited limited interaction profiles, reflecting their specialized architectural functions. Decomposition of outgoing communication patterns at the signaling-pathway level identified myCAFs and fibroblasts as the dominant sources of extracellular matrix and structural signaling (collagen, fibronectin 1, laminin, tenascin, and periostin), while endothelial cells predominantly contributed to vascular-related signaling modules such as PECAM1 and ANGPTL (Figure 7C). Tumor edge cells were the broadest signaling source in the dataset, contributing to a diverse spectrum of pathways, including collagen, FN1, laminin, tenascin, APP, CD99, GAP, MK, and PERIOSTIN, consistent with their role at the invasive front. Incoming communication patterns mirrored this organization: tumor edge populations received diverse matrix- and adhesion-related inputs, reinforcing their position at the tumor-stromal interface, while tumor core cells demonstrated a more limited but coordinated perception of structural and proliferative cues (Figure 7D).

## Discussion

In this work, we introduced CoexpressDeconvolve, a reference-free framework that reconstructs both integer cell numbers and cell type-specific gene expression directly from spot-based ST data. By combining HK gene calibration, a spatial gene co-expression manifold, LDA topic modeling, and hierarchical Bayesian UMI redistribution, the pipeline turns a multicellular Visium slide into a population of discrete single-cell-like profiles compatible with standard scRNA-seq analysis tools.

Benchmarking against three established deconvolution tools placed CoexpressDeconvolve in a specific position in the trade-off landscape. On gene expression reconstruction, it scored within 0.05 Pearson units of cell2location while operating without an external reference and 0.06 Pearson units above STdeconvolve, the standard reference-free tool. On absolute cell counts, it produced predictions roughly 1.5-times as accurate as cell2location’s per-spot estimates and the most concordant per-slide cell-type totals among the methods tested. On cell-type proportion RMSE, CoexpressDeconvolve finished last by a small margin, with the proportion error concentrated on the tumor/dividing tumor pair, which all four methods, including reference-based Tangram, struggled to separate. These results establish CoexpressDeconvolve as the tool of choice when deconvolution of Visium slide gene expression is required without the use of reference scRNA-seq data down to the single-cell and whole transcriptome levels, with slightly reduced accuracy in determining cell type proportions.

Beyond accurate reconstruction, the key practical deliverable of CoexpressDeconvolve is a Space Ranger-compatible HDF5 feature-barcode matrix containing a population of discrete single-cell-like profiles for each slide. This distinguishes it from topic-based reference-free approaches such as STdeconvolve, which infer latent probabilistic topics that require manual interpretation and do not provide a direct interface to standard single-cell analysis workflows. By generating an output that mirrors the format of Space Ranger-derived data, CoexpressDeconvolve enables seamless import into Seurat, Scanpy, and other downstream tools, thereby unlocking the full single-cell analytical ecosystem for Visium datasets. We used this in the tongue squamous cell carcinoma analysis to run trajectory inference (Slingshot and TradeSeq) on the malignant compartment and ligand-receptor communication inference (CellChat) at single-cell-like resolution. The resulting analyses, including the suprabasal keratinocyte-anchored tumor core/tumor edge bifurcation and the identification of the tumor edge as the dominant signaling hub, cannot be run on the raw multicellular Visium spot matrix and would have remained inaccessible under a topic-only deconvolution.

### Limitations of the study

This study has several limitations. The calibration relies on a panel of 15 human HK genes derived from a meta-analysis of 916 single-cell datasets; while the hybrid HK-library-size formulation buffers against transcriptionally extreme regions, the panel itself has not been validated on heavily degraded archival tissue or on tissues with strongly perturbed HK baselines.

In conclusion, CoexpressDeconvolve provides a reference-free path from a single multicellular ST slide to a population of integer-counted, single-cell-like expression profiles. By foregoing the requirement for a paired scRNA-seq atlas, it broadens the set of tissues and pathologies amenable to high-resolution downstream analysis, and the benchmarks reported here delineate the specific regimes in which it should and should not be preferred.

## Resource availability

### Lead contact

Requests for further information and resources should be directed to and will be fulfilled by the lead contact, Olga Perik-Zavodskaia (perik.zavodskaia@gmail.com).

### Materials availability

This study did not generate new unique physical reagents.

### Data and code availability

• This paper analyzes publicly available data. Human breast cancer scRNA-seq and Visium datasets are available at https://www.10xgenomics.com/products/xenium-in-situ/preview-dataset-human-breast (accessed on 08.01.2026); human tongue carcinoma Visium dataset is available at https://doi.org/10.1038/s41598-024-76044-2 (accessed on 08.01.2026).
• All original code is publicly available at the CoexpressDeconvolve GitHub repository (https://github.com/Perik-Zavodskii/CoexpressDeconvolve), has been deposited at Zenodo: https://doi.org/10.5281/zenodo.20777640, and is publicly available as of the date of publication.
• Any additional information required to reanalyze the data reported in this paper is available from the lead contact upon request.

## Acknowledgments

This research was supported by the 10.13039/501100006769Russian Science Foundation, award №25-15-00095 (https://rscf.ru/project/25-15-00095/). The authors thank the 10× Genomics team for making the breast cancer multi-modal preview datasets publicly available and the Association for Single Cell Analysis (ASCA) team for sharing the tongue squamous cell carcinoma Visium dataset at the ASCA 2024 Tomsk single-cell analysis hackathon.

## Author contributions

Conceptualization, S.A., R.P.-Z., and O.P.-Z.; methodology, S.A., R.P.-Z., and O.P.-Z.; software, S.A., R.P.-Z., and O.P.-Z.; formal analysis, S.A., R.P.-Z., and O.P.-Z.; data curation, S.A., R.P.-Z., and O.P.-Z.; visualization, S.A., R.P.-Z., and O.P.-Z.; writing – original draft, S.A., R.P.-Z., O.P.-Z., and S.S.; writing – review and editing, S.A., R.P.-Z., O.P.-Z., and S.S.; supervision, S.S.

## Declaration of interests

The authors declare no competing interests.

## STAR★Methods

### Key resources table

**Table:** 

REAGENT or RESOURCE	SOURCE	IDENTIFIER
**Deposited data**		

Human breast cancer 10x scRNA-seq (3′ chemistry)	10x Genomics	https://www.10xgenomics.com/products/xenium-in-situ/preview-dataset-human-breast
Human breast cancer 10x scRNA-seq (5′ chemistry)	10x Genomics	https://www.10xgenomics.com/products/xenium-in-situ/preview-dataset-human-breast
Human breast cancer 10x Visium spatial transcriptomics (matched section)	10x Genomics	https://www.10xgenomics.com/products/xenium-in-situ/preview-dataset-human-breast
Human tongue squamous cell carcinoma 10x Visium spatial transcriptomics	Patysheva et al.^2^	https://doi.org/10.1038/s41598-024-76044-2
CancerSCEM 2.0 (916 single-cell tumor datasets for housekeeping profile derivation)	Zeng et al.^27^	https://ngdc.cncb.ac.cn/cancerscem/
CoexpressDeconvolve example dataset analysis (this study)	This paper	https://github.com/Perik-Zavodskii/CoexpressDeconvolve/tree/main/Example%20Data

**Software and algorithms**		

CoexpressDeconvolve (Python)	This paper	https://github.com/Perik-Zavodskii/CoexpressDeconvolve; archived release with example configs and analysis scripts
Python 3.10	Python Software Foundation	https://www.python.org/
R 4.4.1	R Core Team	https://www.r-project.org/; RRID: SCR_001905
scanpy	Wolf et al., 2018	https://scanpy.readthedocs.io/; RRID: SCR_018139
Seurat v5.3.1	Hao et al.^28^	RRID: SCR_007322
Harmony	Korsunsky et al.^29^	RRID: SCR_022206
scikit-learn (LDA, FastICA)	Pedregosa et al., 2011	https://scikit-learn.org/; RRID: SCR_002577
umap-learn (UMAP)	McInnes et al.^30^	https://umap-learn.readthedocs.io/
cell2location	Kleshchevnikov et al.^20^	https://cell2location.readthedocs.io/; RRID: SCR_023331
Tangram (tangram-sc)	Biancalani et al.^22^	https://github.com/broadinstitute/Tangram
STdeconvolve	Miller et al.^23^	https://jef.works/STdeconvolve/
StarDist	Schmidt et al.^31^	https://github.com/stardist/stardist; RRID: SCR_022470
Slingshot	Street et al.^32^	RRID: SCR_017012
TradeSeq	Van den Berge et al.^33^	RRID: SCR_021968
CellChat	Jin et al.^26^	RRID: SCR_021946
Matrix (R)	Bates et al.	https://CRAN.R-project.org/package=Matrix
pheatmap (R)	Kolde	https://CRAN.R-project.org/package=pheatmap
viridis / ggplot2 (R)	Wickham, Garnier	https://ggplot2.tidyverse.org/

### Method details

#### Breast cancer scRNA-seq reference atlas processing

The paired 3′- and 5′-chemistry single-cell RNA sequencing libraries released alongside the 10x Genomics human breast cancer Visium preview dataset were merged into a single matrix; cells expressing more than 12,000 genes or with mitochondrial transcript content exceeding 31% were excluded. The integrated dataset underwent standard processing in Seurat v5.3.1^28^ (log-normalization, scaling, PCA), and library-of-origin (chemistry) batch effects were corrected via Harmony^29^ on the principal components. UMAP and Louvain clustering on the Harmony-corrected embedding produced the cell-type-annotated atlas shown in Figure 1, which served as the ground truth reference for the synthetic Visium benchmark and as the reference for the Tangram and cell2location runs.

#### Overview of the CoexpressDeconvolve pipeline

CoexpressDeconvolve is a computational pipeline designed to resolve the cellular heterogeneity of spot-based spatial transcriptomics data by reconstructing single-cell-like expression profiles directly from composite spatial signals, without relying on a paired single-cell RNA-seq reference. The pipeline ingests standard 10x Genomics Visium output files and proceeds through a multi-stage protocol: (1) data reading, (2) approximation of cell counts per spot, (3) biological denoising and feature selection, (4) construction of the spatial gene co-expression manifold, (5) hyperparameter optimization via a perplexity sweep, (6) final deconvolution and manifold-guided topic projection, (7) hierarchical probabilistic sampling, (8) spatial placement of the reconstructed single-cell-like profiles, and (9) data export. All steps are implemented in Python 3.10 and orchestrated by the codeconv.py module distributed in the GitHub repository. The canonical configuration file (codeconv_config.json) is provided in the repository and was used unchanged for every analysis in this study.

#### Obtaining average single-cell parameters

To establish a representative average single-cell profile, we performed a large-scale meta-analysis of 916 single-cell datasets obtained from the Database: CancerSCEM 2.0,^27^ comprising more than seven million tumor, immune, and tumor microenvironment cells. For each dataset, cell-specific library sizes were computed as the total number of unique molecular identifiers (UMIs) per cell. Assuming a Gamma-distributed model of library size variability, a dispersion parameter was estimated by dividing the variance of library sizes by the squared mean within each dataset. To obtain robust global parameters, the per-dataset mean library size (μ = 5,483.7) and the per-dataset dispersion (φ = 0.70) were averaged across all 916 datasets and saved into the canonical engine configuration (codeconv_config.json).

An average housekeeping (HK) gene expression profile was generated in parallel. Within each dataset, raw UMI counts for the curated HK panel (*RPL19, TUBB, EEF1G, PPIA, GAPDH, ABCF1, SDHA, OAZ1, G6PD, ALAS1, GUSB, HPRT1, POLR2A, POLR1B,* and *TBP*) were normalized at the single-cell level by dividing by the corresponding library size and scaling by a factor of 10,000, then log-transformed. The mean log-normalized expression of every HK gene was then computed across all cells in each dataset, and the resulting per-dataset means were averaged gene-wise across the full collection of 916 studies to yield the global HK profile shipped in codeconv_config.json.

#### Step 1: Data reading

The pipeline ingests raw spatial transcriptomic data from the standard output directories generated by the 10x Space Ranger pipeline. Both HDF5 (.h5) and Matrix Market formats are supported for the filtered feature-barcode matrix. Upon loading, the expression matrix is synchronized with the spatial tissue manifest (tissue_positions.csv), which contains the array coordinates and tissue-coverage status for each barcode. Formatting discrepancies between the expression matrix and the spatial manifest are detected and harmonized automatically. After synchronization, the pipeline filters the expression matrix to retain only those barcodes flagged as in_tissue, discarding background spots, and computes the per-spot library depth as a primary quality-control metric.

#### Step 2: Cell count approximation

To transition from relative cell-type proportions to integer cell counts, CoexpressDeconvolve combines two complementary estimators per spot: a housekeeping (HK) gene signal that captures the spot’s transcriptional density relative to a single-cell reference, and a total-library-size signal that captures the spot’s absolute UMI content relative to a slide-wide UMI-per-cell anchor.

##### Housekeeping signal

The pipeline loads the species-specific HK reference shipped in codeconv_config.json and computes the intersection of HK genes present in both the reference and the Visium dataset. For each spot *s*, the raw counts of intersecting HK genes (nHK) are normalized to constant depth (counts per 10,000) and log-transformed; the mean log-normalized HK expression is then computed per spot, and the analogous log-mean is computed for the reference panel. Converting both signals back to linear space yields the HK-derived cell-count estimate:

$$n_{HK}\;\left(s\right)=\exp\;\left[\;mean\left(\log\left({\;HK}_{s}\;\right)\right)\;\right]\;/\;\exp\;\left[\;mean\left(\log\left({\;HK}_{\text{ref}}\;\right)\right)\;\right]\text{,}$$

which represents the cellular content of spot *s* expressed in units of one reference single cell.

##### Library-size signal

In parallel, the pipeline computes a per-cell UMI anchor *U*_per-cell_ by aggregating across all spots that pass the spot-level UMI gate:

$$U_{\text{per}-\text{cell}}{=\Sigma }_{s\in valid}{\;U}_{s}{\;/\;\Sigma }_{s\in valid}{\;n}_{HK}\left(s\right)\text{,}$$

where *U*_*s*_ is the total UMI count of spot *s* and *valid* stands for the spots passing the minimum UMI threshold. The library-size-derived cell-count estimate is then:

$$n_{UMI}\left(s\right)=U_{s}\;/\;U_{\text{per}-\text{cell}}$$

##### Hybrid blend

The two estimates are combined through a weighted geometric mean parameterized by a single slice-level coefficient α ∈ [0, 1]:

$$n\left(s\right)=round\;\left[\;{n_{UMI}\left(s\right)}^{\alpha \;}{{\;\cdot \;n}_{HK}\left(s\right)}^{1-\alpha \;}\right]\text{,}$$

with α = 1.0 reducing to pure library-size calibration and α = 0.0 to pure HK calibration; the default α = 0.6 weights the library-size signal slightly more strongly than the HK signal. The geometric mean to preserve the multiplicative structure of the two ratio-based estimates and bounds the hybrid estimate by the smaller of the two factors, which is the behavior required at the two regime boundaries: in transcriptionally depleted regions (necrosis, edge artifact), the small *n*_UMI_ factor pulls the estimate toward zero even when residual housekeeping transcripts are present; in highly proliferative regions, the large *n*_UMI_ factor prevents the HK signal from under-counting cells driven into transcriptionally hyperactive states.

##### Quality control

Low-quality spots are identified by a single user-defined absolute UMI floor *U*_min_ (default 300; we recommend setting this near the first percentile of the in-tissue spot UMI distribution shown on the per-slice QC plot). Spots with *U*_*s*_ < *U*_min_ are set to *n*(*s*) = 0 and excluded from all downstream steps. The threshold is exposed as the min_umi parameter in the configuration.

#### Step 3: Denoising and feature selection

To maximize the signal-to-noise ratio for downstream probabilistic modeling, the pipeline first removes non-informative or technically confounded transcripts using a species-specific regular expression that matches mitochondrial genes, cytosolic ribosomal proteins, long intergenic non-coding RNAs, microRNA precursors, and pre-mRNA / unannotated transcript artefacts. The noise-filtered gene list is retained as the working gene set and is the input to the downstream manifold and projection steps. To build the LDA training corpus, the pipeline then follows STdeconvolve’s^23^ restrictCorpus logic: the spot-presence fraction (the fraction of spots in which a gene has non-zero counts) is computed for every gene using sparse-matrix operations, and a gene is admitted to the candidate pool only if its presence fraction lies in [0.05, 0.95], excluding both rarely-detected genes and ubiquitously expressed genes that carry no spot-level information. Over-dispersed genes are then selected from this candidate pool via mean-variance trend residuals. Counts are library-size normalized to 10,000 and log1p-transformed (Log1p CP10K); per-gene means and variances are computed directly on the sparse matrix, so the spatial matrix is never densified. A smoothed cubic polynomial trend log10(var) ∼ poly3(log10(mean)) is fit across the candidate genes, and each gene’s residual above the fitted trend is used as its overdispersion score. The top 3,000 genes by residual are retained as the over-dispersed gene set and serve as the training corpus for LDA topic modeling. Genes excluded by the noise regex or the presence filter remain available to subsequent steps so that manifold construction (Step 4) and the topic-gene projection (Step 6) still extend the deconvolution to the full transcriptome.

#### Step 4: Construction of the spatial gene co-expression manifold

The pipeline transposes the expression matrix to cluster genes by their spatial co-occurrence. The gene expression matrix is log-transformed and unit-variance scaled. To disentangle the mixed transcriptional signals inherent to multicellular spots, the pipeline employs FastICA^34^; unlike PCA, ICA maximizes non-Gaussianity to isolate statistically independent biological sources. The resulting independent components are projected into a low-dimensional latent space using UMAP^30^ with a cosine distance metric. This places co-expressed genes as geometric neighbors in the latent space, and the resulting topology serves as the scaffold for imputing non-ODG genes in Step 6.

#### Step 5: Hyperparameter optimization via perplexity sweep with rare-topic gate

To determine the optimal number of latent topics K, the pipeline runs a sweep over a user-defined range (default 3-15). For each K, the pipeline trains an LDA model on the ODG matrix, computes held-out (70/30 split is used) perplexity (lower = better fit), and counts the number of rare topics defined as topics with mean spot-prevalence $\bar{\theta }$ below a threshold (default 0.05). The default model selection rule is to choose the smallest K minimizing perplexity that does not introduce more than one rare topic; the rare-topic gate prevents over-parameterized models that split coherent cellular programs into low-prevalence fragments. Both the perplexity curve and the rare-topic count are reported (Figure S4). This procedure provides explicit sensitivity to the topic-number choice and an interpretable stability criterion.

#### Step 6: Final deconvolution and manifold-guided topic projection

Following K selection, the pipeline trains a definitive LDA model on the ODG matrix to learn the spot-topic distribution matrix *θ* (whose rows are the per-spot topic prevalence vectors) and the topic-gene distribution matrix *β* over ODGs. To recover genome-wide topic-gene weights, the topic definitions are then projected onto the whole-transcriptome manifold constructed in Step 4. For each non-ODG gene *g*, let KNN(*g*) denote its k-nearest ODG neighbors in the FastICA-UMAP latent space and *c*_*g,i*_ (their cosine similarity in that space). The projected topic-gene weight is the cosine-similarity-weighted average of the ODG-side β over the gene’s neighbors:

$$\beta_{k,g}=\left(\;\Sigma_{i\in KNN\left(g\right)}{\;c}_{g,i}{\;\cdot \;\beta }_{k,i}\;\right)\;/\;\left(\;\Sigma_{i\in KNN\left(g\right)}{\;c}_{g,i}\;\right)\text{.}$$

Genes whose maximum cosine similarity to any ODG anchor falls below a low-confidence floor (default 0.05) (Figures S5 and S6) are not assigned a sharp topic profile; their UMIs are instead distributed uniformly across the reconstructed single-cell-like profiles of the spot (Step 7).

#### Step 7: Hierarchical probabilistic sampling

Continuous topic probabilities are transformed into discrete single-cell-like profiles through a four-stage hierarchical sampling that conserves the observed per-spot UMI marginals. Let *N*_*s*_ be the integer cell count of spot *s* (Step 2), *θ*_*s*_ its topic prevalence vector (Step 6)—that is, the *K*-dimensional vector *θ*_*s*_ = (*θ*_*s,1*_, …, *θ*_*s,K*_) ∈ ℝ^*K*^, with *θ*_*s,k*_ ≥ 0 and Σ_*k=1*_^*K*^ *θ*_*s,k*_ = 1, where *K* is the number of LDA topics (Step 5), and *U*_*s,g*_ the observed UMI count of gene *g* in spot *s*. The sampler proceeds as follows.

##### Cell-to-topic assignment

Cells in the spot are partitioned among the K topics by a single multinomial draw,

$$\left(\;m_{s1}{,\ldots ,m}_{\text{sK}}\;\right)\;∼\;Multinomial\left(\;N_{s},\theta_{s}\;\right)\text{,}$$

where *m*_*s,k*_ is the number of single-cell-like profiles assigned to topic *k* in spot *s* and Σ_*k=1*_^*K*^ *m*_*s,k*_ = *N*_*s*_.

##### Per-cell RNA-content weight

To model biological heterogeneity in cellular RNA content, each generated cell *c* receives an independent Gamma-distributed weight

$$w_{s,c}\;∼\;Gamma\left(\;shape=1\;/\;\varphi ,scale=\mu \;\cdot \;\varphi \;\right)\text{,}$$

with engine parameters (μ, φ) - the global mean and dispersion of single-cell library sizes - provided by codeconv_config.json. This ensures that var(w) / E[*w*]^2^ matches the empirically observed cell-level UMI dispersion across the CancerSCEM 2.0 meta-analysis.

##### UMI-to-topic allocation

For each gene *g* detected in spot *s*, the *U*_*s,g*_ observed UMIs are partitioned among topics by multinomial sampling with Bayes-rule probabilities,

$$P\left(\;topic=k\;|\;g,s\;\right)=\beta_{k,g}{\;\cdot \;\theta }_{s,k}{{\;/\;\Sigma }_{k^{\prime }=1}}^{K}{\;\beta }_{k^{\prime },g}{\;\cdot \;\theta }_{s,k^{\prime }}\text{,}$$

yielding the per-topic UMI counts *u*_*s,g,k*_.

##### UMI-to-cell allocation within a topic

The *u*_*s,g,k*_ UMIs of gene *g* assigned to topic *k* are further distributed among the *m*_*s,k*_ cells of that topic by a second multinomial draw with cell probabilities proportional to their Gamma weights:

$$P\left(\;cell=c\;|\;topic=k,g,s\;\right)=w_{s,c}{\;/\;\Sigma }_{c^{\prime }\in topic\;k}\;w_{s,c^{\prime }}$$

For genes flagged as low-confidence in Step 6 (maximum ODG-anchor cosine similarity below the floor), step (iii) is bypassed, and the gene’s UMIs are distributed uniformly across the *N*_*s*_ cells of the spot, contributing only background, cell-type-agnostic signal. The sampler conserves the per-spot, per-gene UMI marginals by construction.

#### Step 8: Spatial placement

To enable spatially aware downstream analysis, the generated single cells are assigned physical coordinates within the capture area of their parent spot. The pipeline uses a Fibonacci spiral (golden-angle) lattice to distribute cells uniformly from the spot center outward; this deterministic packing keeps every reconstructed cell within the Visium fiducial diameter and ensures visibility of individual single-cell-like profiles on spatial plots.

#### Step 9: Data export

The reconstructed dataset is exported in a Space Ranger-compatible structure: a filtered feature-barcode HDF5 matrix (.h5) containing the single-cell-like profiles, paired with a spatial directory containing tissue images, scale factors, and a synchronized tissue_positions.csv. This architecture allows the deconvolved data to be loaded directly into standard analysis environments, including Seurat’s Load10X_Spatial function, without custom parsers.

#### Synthetic Visium dataset construction

The synthetic Visium dataset used for benchmarking was generated from the integrated breast cancer scRNA-seq atlas. A 50 × 50 hexagonally offset grid (2,500 spots) was constructed in Seurat (v5.3.1); each spot was assigned array and pixel coordinates and a unique synthetic barcode compatible with the 10x feature-barcode matrix convention. The number of cells contributing to each spot was sampled from a truncated normal distribution (mean 5, SD 3, constrained between 1 and 10 cells), and for each spot, the corresponding number of single cells was sampled with replacement from the full atlas; their raw UMI vectors were summed gene-wise to produce a composite spot-level profile. The resulting gene-by-spot matrix and the per-spot per-cell-type cell counts were exported as an HDF5 file with placeholder H&E images, structurally identical to standard Space Ranger outputs, and made available alongside the benchmark notebooks in the repository.

#### Competing methods

All benchmarks used a single, frozen list of ODGs per slide, so that every method received the same gene set as input.

##### Tangram

Implemented via tangram-sc with PyTorch. The integrated scRNA-seq atlas was used as the reference, with the CellType column as the cell-type label. After tg.pp_adatas alignment on the curated ODGs, mapping was run with tg.map_cells_to_space using mode=“cells”, density_prior=“rna_count_based”, and CPU acceleration; the projected whole-transcriptome expression was obtained via tg.project_genes. In cells mode, each ad_map row sums to ∼1, so the per-cell-type pseudobulk reduces to the reference atlas’s own per-CellType UMI pseudobulk, which explains the perfect Tangram correlations observed in our benchmark.

##### cell2location

Implemented via the official cell2location Python package. Reference signatures were learned with RegressionModel.setup_anndata(labels_key=“CellType”) and trained on the ODG-subsetted atlas with max_epochs=250, batch_size=1000, train_size=1, lr=0.002; the posterior was exported with num_samples=1000. Spatial deconvolution was then run with Cell2location(N_cells_per_location=5), max_epochs=15000, train_size=1, accelerator=“cpu”, and num_samples=1000. Per-spot cell-type abundance was taken as the q05_cell_abundance_w_sf posterior summary and rounded to non-negative integers to enable direct comparison with CoexpressDeconvolve cell counts; proportions were computed from the original continuous abundance.

##### STdeconvolve

Implemented in R using the STdeconvolve package, with set.seed(42). The ODG-subsetted spot count matrix was used as the LDA corpus, and the topic number was manually fixed at K = 12 for the synthetic dataset. Topics were correlated with the scRNA-seq atlas pseudobulk by Pearson correlation, and a topic-to-cell-type mapping was assigned manually based on the resulting correlation heatmap. Per-cell-type GEX was reconstructed by scaling the topic β vectors by the per-topic UMI mass (Σ_spots θ_{spot,k} x spot_UMI_ODG) and summing across topics assigned to the same cell type.

#### Nuclear segmentation by StarDist

To quantify the per-spot relationship between captured UMI and image-based nuclear counts, we ran StarDist^31^ on the H&E section of the tongue squamous cell carcinoma slide, using the pretrained 2D Versatile (H&E) model and default thresholds. Detected nuclei centroids were assigned to the nearest Visium spot center, and the per-spot nuclear count was correlated with the captured UMI per spot. The obtained results were also compared with the relationships between UMI per spot counts and the real and CoexpressDeconvolve-detected cells (Figures S7–S9).

#### Downstream analysis of deconvolved Visium data

For both the tongue cancer and breast cancer datasets, the deconvolved HDF5 outputs were loaded into Seurat v5.3.1^28^ via Load10X_Spatial. Raw counts were normalized and variance-stabilized via LogNormalize and ScaleData. PCA on the variable features was followed by UMAP, neighbor graph construction, and Louvain clustering. Cluster identity assignments used canonical marker genes (visualized via DotPlot). Spatial mapping of the assigned identities used SpatialDimPlot.

#### Pseudotime and trajectory inference

To model the spatial and transcriptional progression of tumor invasion in tongue cancer, trajectory inference was performed on the deconvolved Suprabasal Keratinocytes plus the malignant compartment using the Slingshot R package.^32^ The Seurat object was subsetted to Suprabasal Keratinocytes, Tumor Core, Tumor Front, and Tumor Edge. Lineages were inferred from UMAP embeddings via getLineages with Suprabasal Keratinocytes set as the starting cluster, allowing the algorithm to identify the bifurcating endpoints. Principal curves were fitted via getCurves. To identify genes with dynamic expression patterns along the trajectory, Negative Binomial Generalized Additive Models (GAMs) were fitted on the raw count matrix using the fitGAM function from TradeSeq.^33^ Differential expression along the trajectory was assessed with startVsEndTest under FDR correction; smoothed profiles were generated with predictSmooth, and log2 fold-changes were computed between terminal and starting points. Trajectory markers were defined by adjusted *p* < 0.05 and absolute log2FC > 0.5; expression along pseudotime was visualized with ggplot2 with GAM-smoothed trendlines per lineage.

#### Intercellular communication network

The CellChat R package^26^ was applied to the deconvolved tongue squamous cell carcinoma Seurat object. The CellChatDB.human ligand-receptor database was used to model signaling probabilities. Aggregate interaction strength was computed across all defined cell types with computeCommunProb and computeCommunProbPathway, and dominant signaling roles were identified by netAnalysis_signalingRole_scatter. Outgoing and incoming communication patterns at the pathway level were summarized as dot plots, with dot size encoding relative pathway-level contribution. Spatial co-localization was computed as the pairwise positivity score between cell types after CoexpressDeconvolve placement.

### Quantification and statistical analysis

#### Reconstruction fidelity

For each method, we computed per-cell-type pseudobulk expression profiles by summing UMIs (or weighted UMIs for ad_map / β-weighted methods) within each predicted cell type. We then correlated these pseudobulks with the matched scRNA-seq atlas pseudobulks computed identically (sum of UMIs within each labeled cell type). Pearson correlation coefficients and Lin’s Concordance Correlation Coefficients (CCC) were computed across 3,000 ODGs for the synthetic benchmark and across 3,500 highly variable genes for the real breast cancer slide.

#### Cell count and proportion accuracy

For methods returning integer per-spot cell counts (CoexpressDeconvolve, cell2location), we computed Pearson correlation, CCC, and RMSE between predicted and ground-truth per-spot total cell counts. For all four methods, we additionally computed RMSE between predicted and ground-truth per-cell-type proportions.

#### Software versions and reproducibility

All Python analyses used Python 3.10, with scanpy, scikit-learn (LDA, FastICA), umap-learn, tangram-sc, and cell2location. All R analyses used R 4.4.1 with Seurat v5.3.1, Harmony, STdeconvolve, Slingshot, TradeSeq, and CellChat. STdeconvolve runs used set.seed(42) explicitly. For cell2location, the q05 posterior summary was used as the abundance estimator throughout. FDR correction used the Benjamini-Hochberg method throughout.
