# Supplementary material for: CoexpressDeconvolve enables reference-free single-cell-resolution deconvolution from spot-based spatial transcriptomics
Source: iScience. 2026 Sep 1;29(9):116824. doi: 10.1016/j.isci.2026.116824 (PMC13571154; doi:10.1016/j.isci.2026.116824)
Supplement: Supplementary file 1 [file mmc1.pdf]

iScience, Volume *29*

Supplemental information

CoexpressDeconvolve enables reference-free single-cell-resolution deconvolution from spot-based spatial transcriptomics

Olga Perik-Zavodskaia, Roman Perik-Zavodskii, Saleh Alrhmoun, and Sergey Sennikov

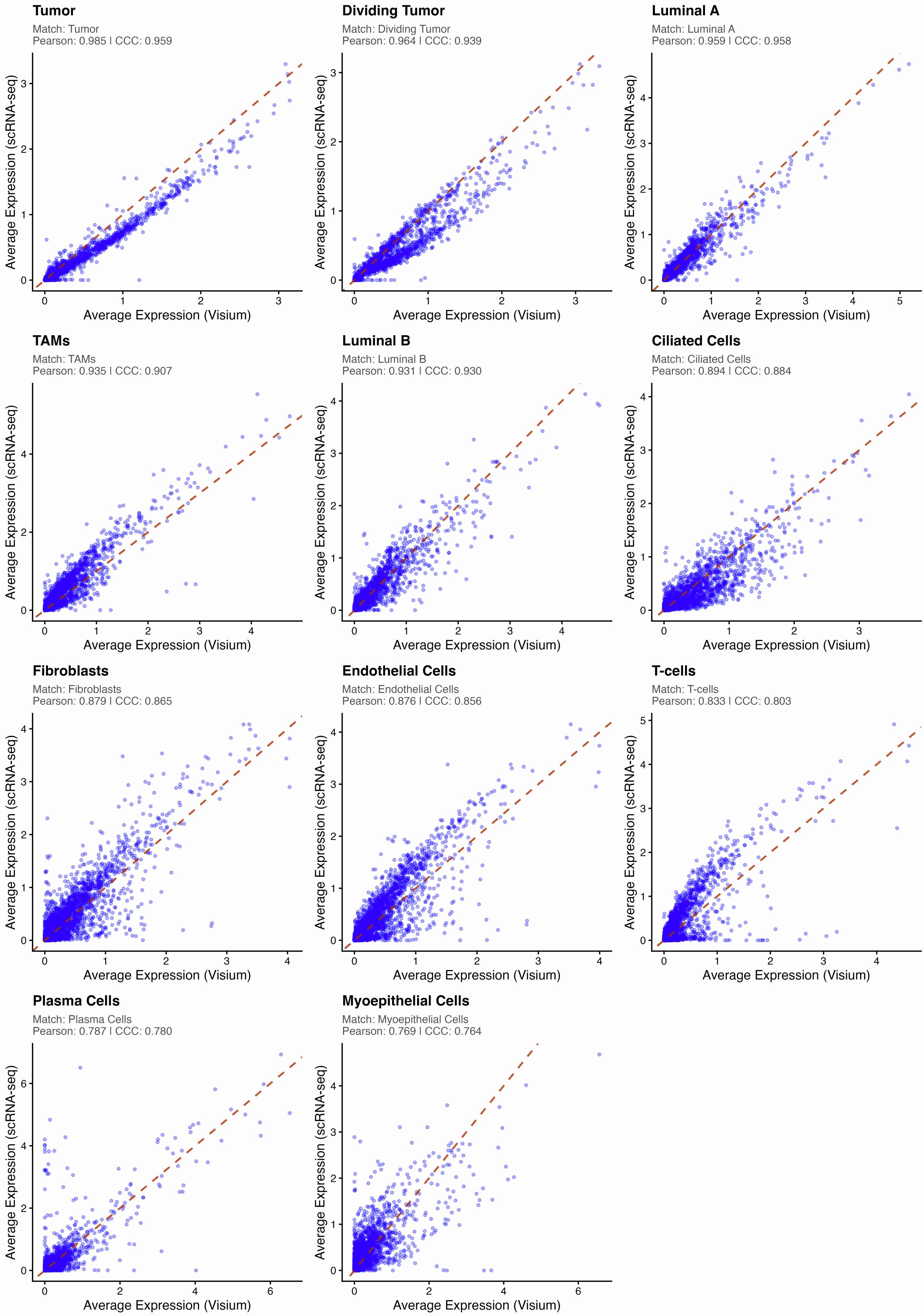

Supplementary Figure 1. Scatterplots comparing Visium synthetic spatial transcriptomics gene expression data derived from scRNA-seq with real scRNA-seq data (3.500 highly variable genes), supplemented with Pearson correlation coefficient and Lin concordance correlation coefficient (CCC) values.

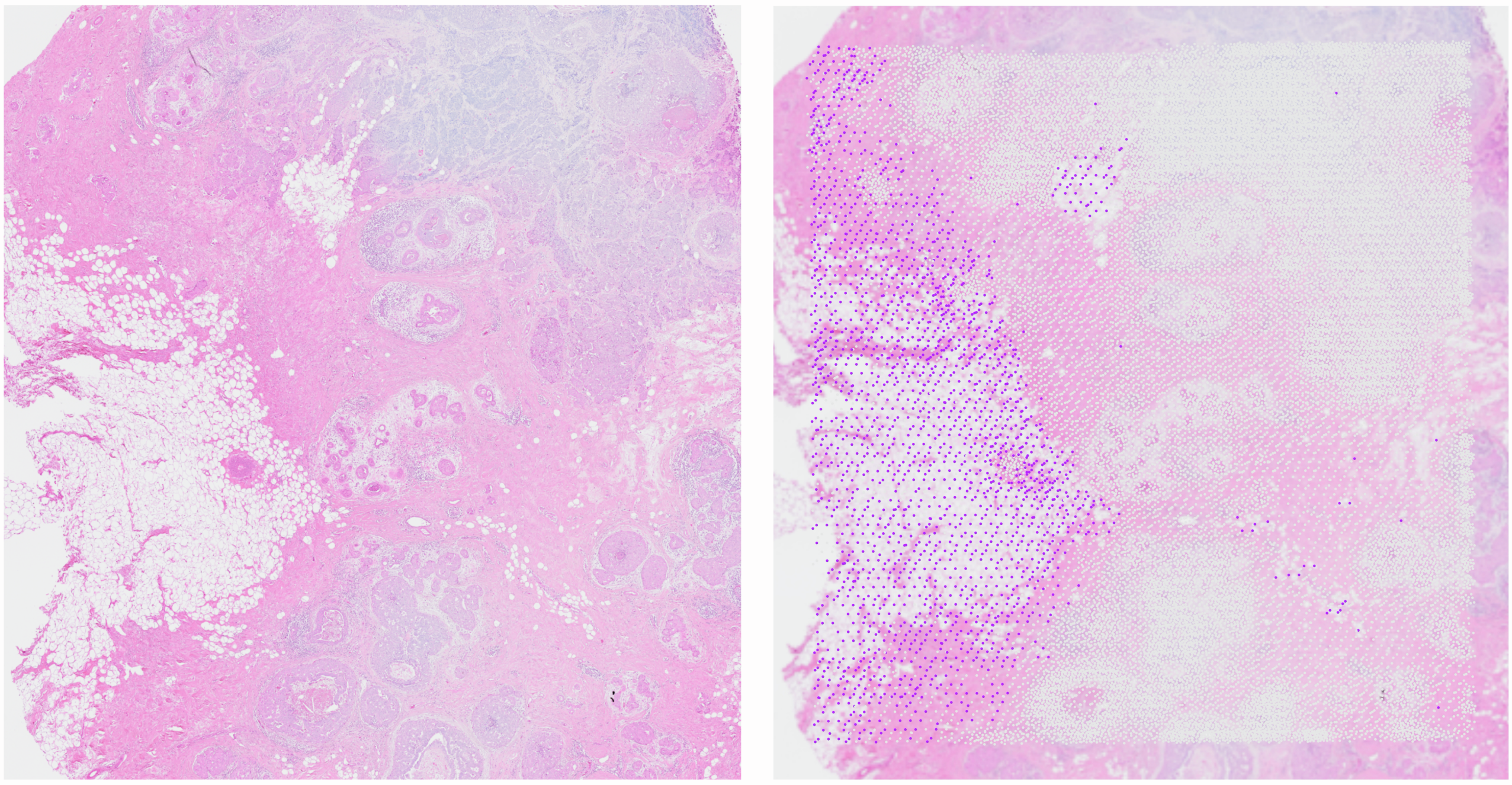

Supplementary Figure 2. An image of adipocytes in the breast cancer dataset obtained using hematoxylin and eosin (H&E) and Visium (CoexpressDeconvolve); adipocytes are shown as nearly empty tissue on the left in the H&E image (left) and highlighted in purple in the spatial cell plot (right).

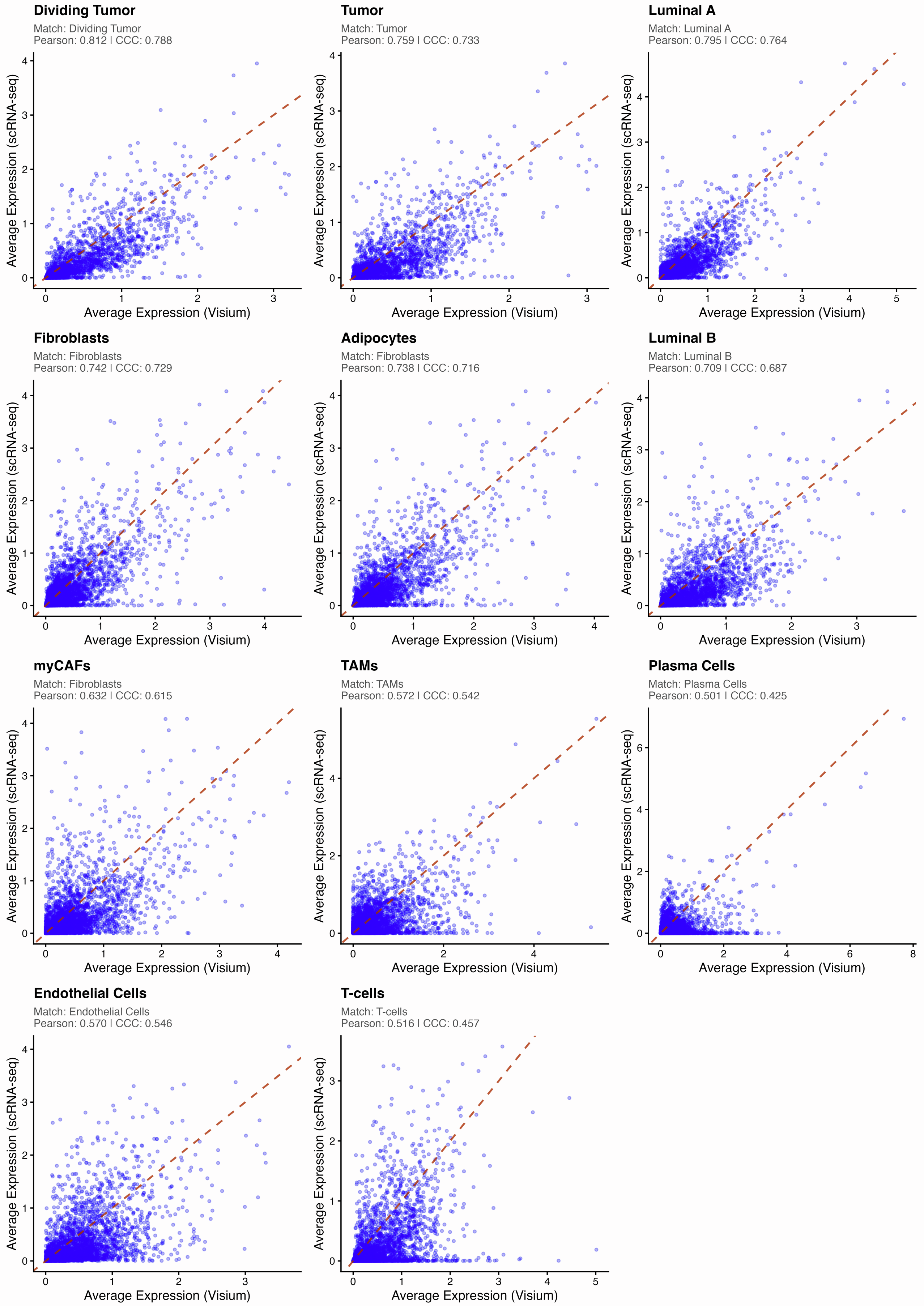

Supplementary Figure 3. Scatterplots comparing real Visium spatial transcriptomics gene expression data with real scRNA-seq data (3.500 highly variable genes), supplemented with Pearson correlation coefficient and Lin concordance correlation coefficient (CCC) values.

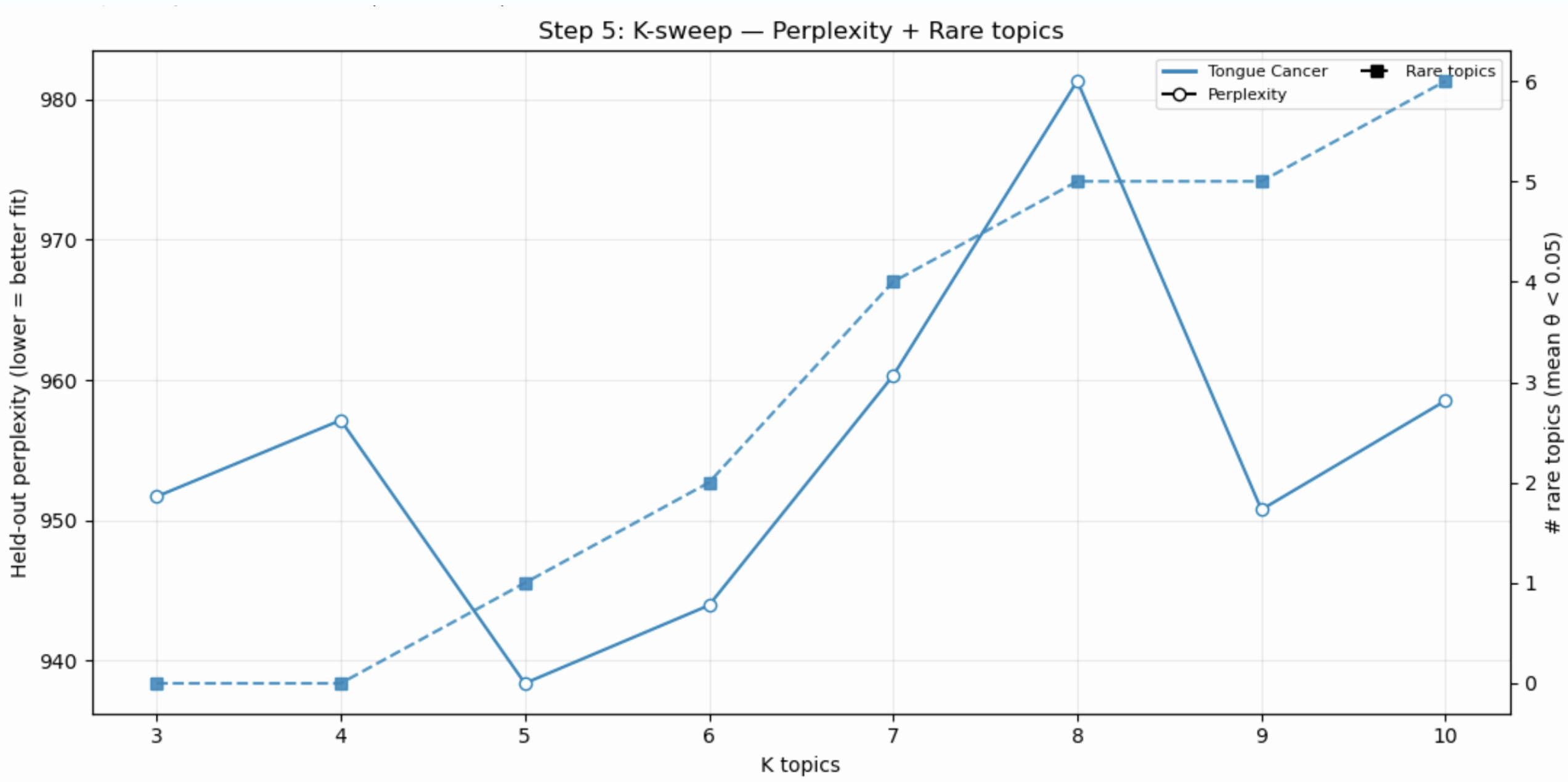

Supplementary Figure 4. K-sweep and rare-cell-type sweeps for optimal K selection.

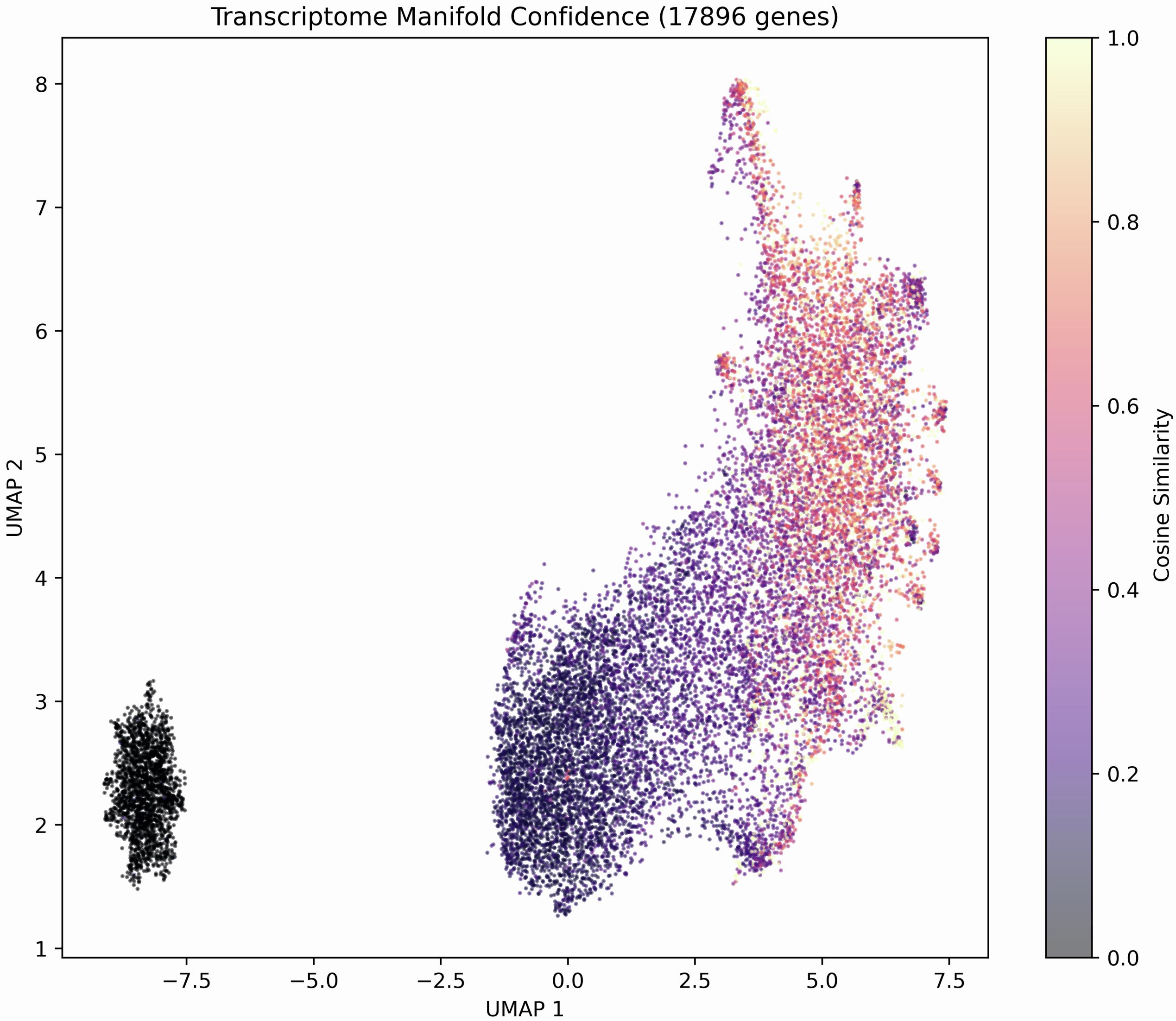

Supplementary Figure 5. Gene co-expression UMAP plot colored by cosine distance with the overdispersed genes. Cluster colored in black represents noise genes with no co-expression to the overdispersed genes.

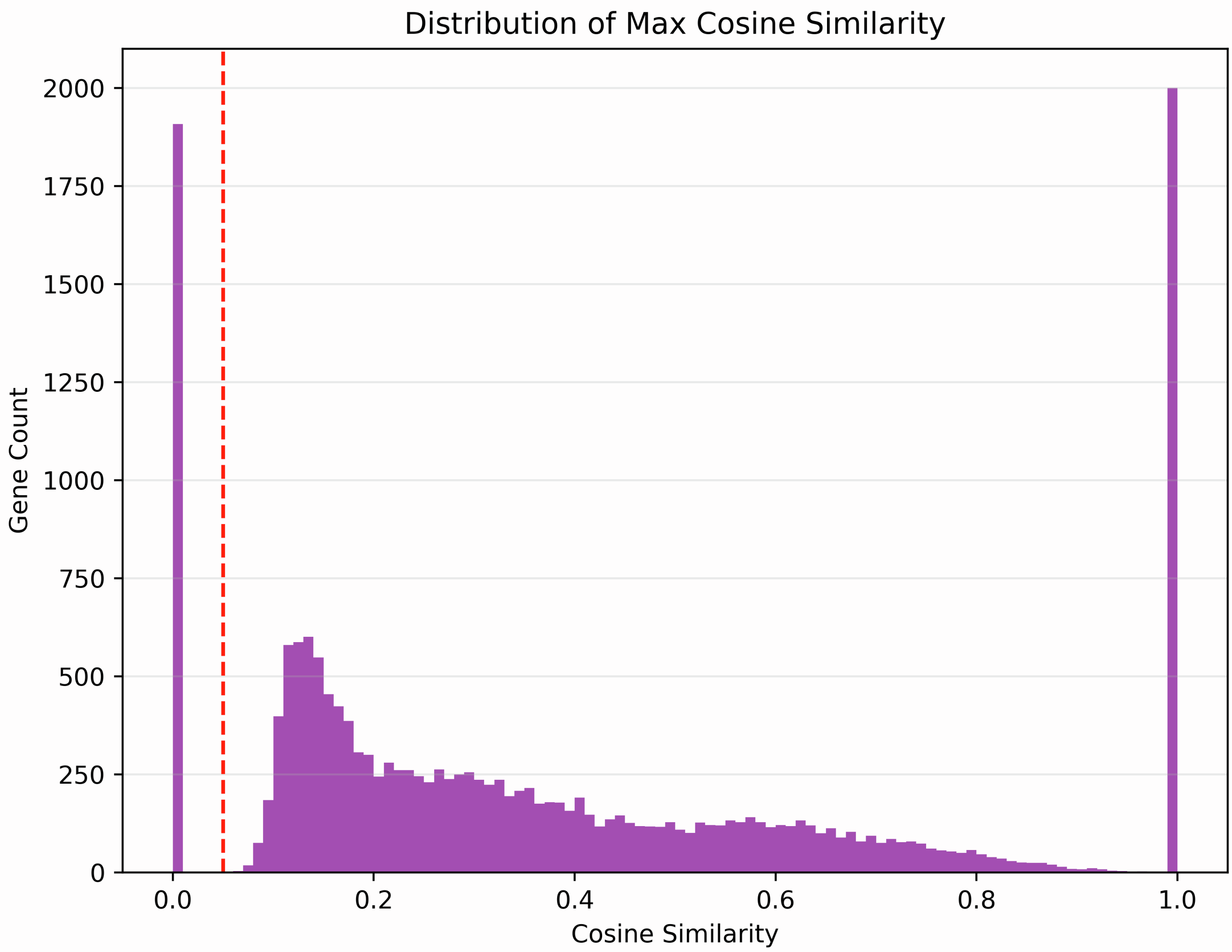

Supplementary Figure 6. Gene co-expression bar plot of cosine distances to the overdispersed genes. The vertical red line represents the noise gene gate.

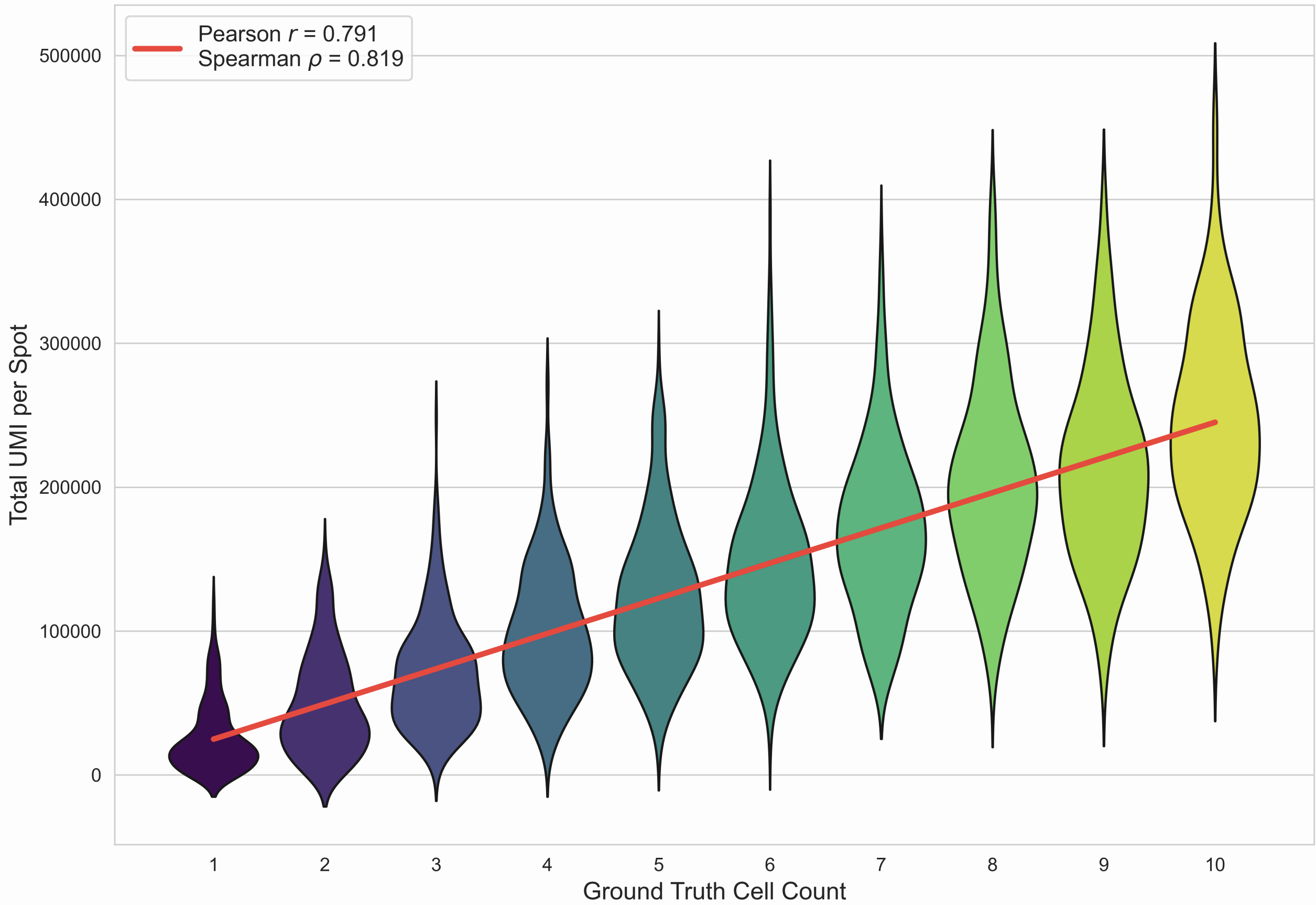

Supplementary Figure 7. Violin plots of the ground truth cells in the *in silico* Visium data versus the per-spot UMI counts.

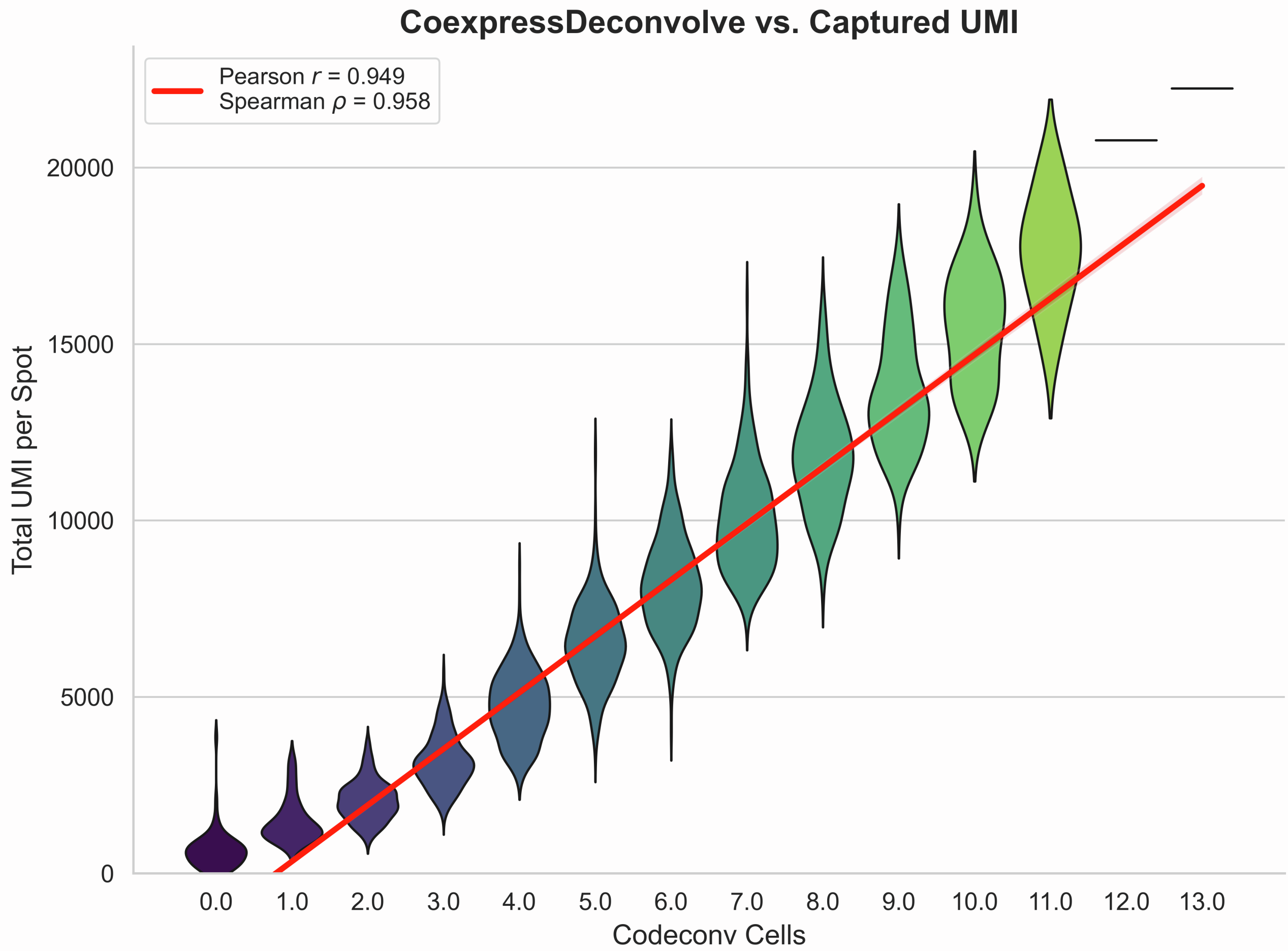

Supplementary Figure 8. Violin plots of the CoexpressDeconvolved-detected cells in the Tongue cancer dataset versus the per-spot UMI counts.

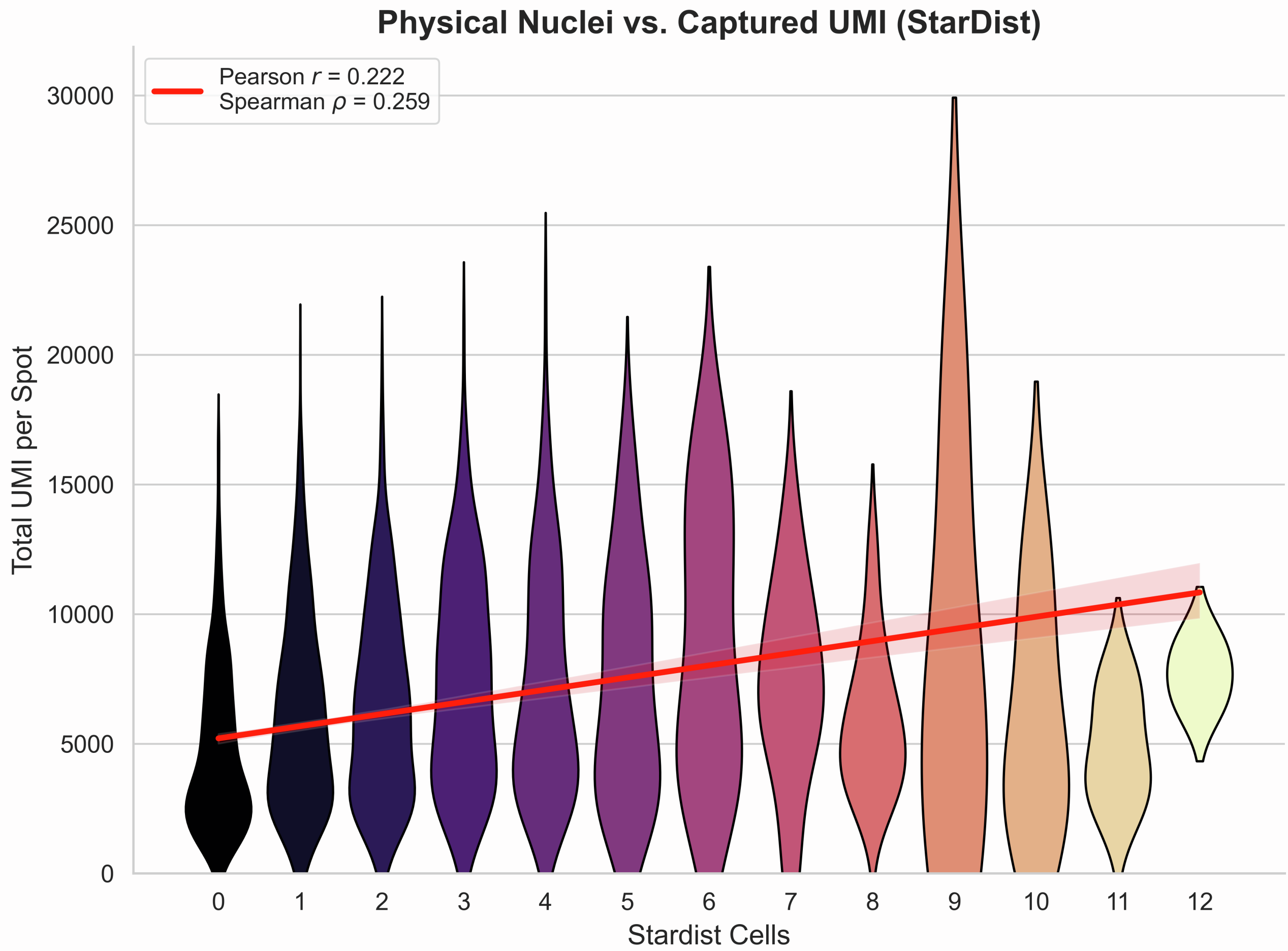

Supplementary Figure 9. Violin plots of the StarDist-detected cells versus the per-spot UMI counts (Tongue cancer dataset).
